# Gene Expression Noise Enhances Robust Organization of the Early Mammalian Blastocyst

**DOI:** 10.1371/journal.pcbi.1005320

**Published:** 2017-01-23

**Authors:** William R. Holmes, Nabora Soledad Reyes de Mochel, Qixuan Wang, Huijing Du, Tao Peng, Michael Chiang, Olivier Cinquin, Ken Cho, Qing Nie

**Affiliations:** 1 Department of Physics and Astronomy, Vanderbilt University, Nashville TN, United States of America; 2 Center for Complex Biological Systems, University of California, Irvine, Irvine, CA, United States of America; 3 Department of Developmental and Cell Biology, University of California, Irvine, Irvine, CA, United States of America; 4 Department of Mathematics, University of California, Irvine, Irvine, CA, United States of America; University of Michigan Medical School, UNITED STATES

## Abstract

A critical event in mammalian embryo development is construction of an inner cell mass surrounded by a trophoectoderm (a shell of cells that later form extraembryonic structures). We utilize multi-scale, stochastic modeling to investigate the design principles responsible for robust establishment of these structures. This investigation makes three predictions, each supported by our quantitative imaging. First, stochasticity in the expression of critical genes promotes cell plasticity and has a critical role in accurately organizing the developing mouse blastocyst. Second, asymmetry in the levels of noise variation (expression fluctuation) of Cdx2 and Oct4 provides a means to gain the benefits of noise-mediated plasticity while ameliorating the potentially detrimental effects of stochasticity. Finally, by controlling the timing and pace of cell fate specification, the embryo temporally modulates plasticity and creates a time window during which each cell can continually read its environment and adjusts its fate. These results suggest noise has a crucial role in maintaining cellular plasticity and organizing the blastocyst.

## Author Summary

A critical event in mammalian embryo development is construction of a mass of embryonic stem cells surrounded by a distinct shell that later forms the placenta along with other structures. Despite sustained investigation, multiple hypotheses for what is responsible for this organization persist and it remains unclear what is responsible for the robust organization (remarkable ability for embryos to pattern correctly) of these structures. Here, we utilize multi-scale, stochastic modeling along with fluorescence imaging to investigate the factors that contribute to the incredible robustness of this organizational process. Results point to two factors that contribute to this robustness: 1) the timing and pace of cell fate specification and 2) stochastic gene regulatory effects. The former creates a window of time during which each cell can continually read their environment and adjust their gene expressions (and consequently fate) in response to dynamic rearrangements of cells arising from cell divisions and motions. The latter improves cell plasticity, providing the capability for cells to adjust to changes in their local environment. Fluorescence imaging results demonstrate that the magnitude and structure of gene expression variations match those predicted to promote organizational robustness.

## Introduction

A central question of developmental biology is how a single cell gives rise to an organism of exquisite complexity. In mammals, the fertilized egg begins this process by dividing multiple times to form a morula, which then undergoes compaction to create the blastocyst. Each cell of the early cleavage stage embryo is considered to be totipotent. After compaction, these cells differentiate to become either the inner cell mass (ICM), which mainly gives rise to the future embryo, or the trophectoderm (TE), which forms extra-embryonic structures. This lineage divergence is the first differentiation event in mammalian development, and is also an intensely studied process in mammalian reproductive biology [1, 2].

**Fig 1:**
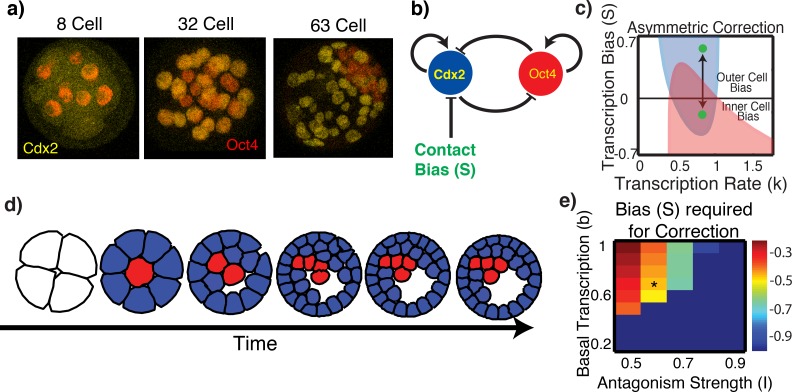
Contact mediated control of Cdx2 transcription is insufficient for proper TE / ICM specification on its own. ***a)*** Images showing the localization of Oct4 / Cdx2 at different embryonic stages. ***b)*** Schematic of transcriptional interactions. ***c)*** State space showing the possible expression states as a function of *k* and the bias *S*. The red region represents Oct4 (ICM) and the blue represents Cdx2 (TE) dominant states. In the overlap, the system is bistable, emitting both TE and ICM states. The two green dots indicate two cell fate states a cell can transition. This correspond to an asterisk indicated in panel (e). ***d)*** Simulation snapshots showing the evolution of the embryo subject to contact signaling. Coloring of cells indicates the dominant factor present (blue = Cdx2 and red = Oct4, matching panel b). Results show a number of interior cells expressing TE factors. ***e)*** The minimum bias (*S*_*i*_) that guarantees a TE to ICM fate switch for different values *I* and *b*, measured as the minimum value of the blue curve in panel *c*. Dark blue indicates that a change of bias alone cannot drive fate correction. The black asterisk denotes parameters derived from published data [9] and used in subsequent analysis.

ICM and TE cell populations are distinguished by both their spatial position within an embryo and gene expression differences. Structurally, the ICM is located in the interior of the blastocyst and the TE forms an outer layer surrounding it. Investigations have revealed that polarity of cells along with cleavage orientation of cell division affect development of this structure [3–6]. Molecularly, Pou5f1/Oct4 (abbreviated Oct4 hereafter), Nanog, and Sox2 transcription factors (TFs) specify ICM cells, while Tead4 and Cdx2 TFs specify the TE [1, 7] (Fig 1A). Interplay among these TFs is critical in specifying the ICM and TE cell fates [2, 5, 8, 9]. These findings imply that a preimplantation mouse embryo interprets various types of information and coordinates the cellular response to produce a normal blastocyst.

Although lineage biasing of blastomeres might start as early as the 4-cell stage [3, 4, 10–14], the embryo is a dynamic entity and each cell at this stage maintains the ability to give rise to both embryonic and extra embryonic tissues [15]. Removal of a blastomere(s) of the cleavage stage embryo can accommodate the loss of cells and still generate normal embryos [16]. Additionally, the formation of chimeric mice can be accomplished by fusing cleavage stage embryos [17, 18]. These manipulated embryos compensate for the changes in spatial rearrangement, cell-cell interactions, and the number of cells, leading to generation of normal newborns [16, 19]. The extraordinary adaptability and robustness of the system begs the question of what mechanism governs the earliest cell lineage decision-making process in mammals.

Time-lapse microscopy has been used to study lineage allocation in unmanipulated mouse embryos [20–22]. Bischoff et al. [20] showed that 95% of cells maintained their position after the 32-cell stage, and the identity of the remaining 5% of cells was unknown. Independently, by carefully following the movement of each cell between 8-32-cell stages, Watanabe et al. [21] found that an average of 1.5 cells per embryo move away from the surface of the embryo toward inside during the segregation of ICM and TE cells [21]. These dynamic cell movements suggest that a robust organizational mechanism must exist for both constructing the proper embryonic architecture, and correcting organizational mistakes that arise over time from stochastic motion and division of cells.

Three hypotheses have been proposed to explain the mechanism underlying the differentiation of totipotent blastomeres cells into the ICM and TE lineages. The “prepattering hypothesis” takes into account the lineage bias that cleavage patterns of blastomeres at the 2-cell embryo (equatorial vs mariginal) establish ICM or TE bias at the 4-cell stage [3, 4, 23, 24]. Consistent with the model, Cdx2 mRNA was shown to be asymmetrically localized in the cytoplasm by the 8-cell stage [5, 25]. In addition, differential localization of Oct4 can be achieved by the 8-cell stage, via differential nuclear export, which results in developmental heterogeneity [26]. While these findings provide some molecular evidence supporting the prepatterning hypothesis, maternal expression of Oct4 and Cdx2 does not seem to be essential for early mouse development [27–29]. The “cell polarity hypothesis”, which posits that polarity along with regulation of the angle and type of cell division organizes the embryo, is supported by the observation that externally localized cells show apical-basal polarity [30, 31] by the 8~16 cell stage. These cells can divide either asymmetrically to produce both TE and ICM daughter cells [6, 30], or symmetrically to generate daughter cells that both become TE. Consistent with this model, loss of the apical complex component Par3 preferentially directs cells to contribute to the ICM lineage [32].

The third hypothesis, the “inside-outside” model, posits that ICM and TE cell fate depends on the position of cells after the 16-cell stage. Experimentally, this was demonstrated by showing that upon removal of TE cells by immunosurgery, ICM cells positioned on the outside of the ICM acquired TE identity [33, 34]. The finding supports the notion that inside-outside position is sufficient to determine TE and ICM identity, and that ICM cells constantly sense surrounding positional information. Additionally, it was experimentally demonstrated that cell-cell contact initiates a signaling cascade through the YAP / Hippo / Tead4 pathway that influences Cdx2 expression [35–38]. This supports the notion that differences in cell-cell contact between inside and outside cells leads to differential TF expression that influences cell fate. Despite the distinct differences between these hypothesized mechanisms, we note that they are not mutually exclusive. Rather, all three are likely to contribute to some extent to maintaining embryonic organization.

A number of modeling works have been carried out to determine the efficacy of different hypotheses. Previous work [39] suggests that mechanical forces alone could give rise to the morphologically distinct structure of interior and exterior cells (e.g. more rounded versus elongated). This investigation, however, modeled only the physical structure of the embryo and did not account for gene expression dynamics. The interactions between early embryonic TFs associated with TE and ICM lineages have been studied independently of the spatial structure of the embryo [40, 41]. More recently, mechanics and gene regulation were combined in a single model [42] to investigate embryonic organization, where it was first shown that the polarity hypothesis may aid organization but is not sufficient to ensure robust patterning. This model however did not incorporate quantitative gene expression data or consider stochastic effects.

Here, we develop a more comprehensive computational modeling platform to investigate this organizational process and test the potential influence of different processes such as cell-cell contact based information, molecular pre-patterns, and stochastic effects. Given the likely large number of contributing influences on organization, rather than attempt to account for all aspects of blastocyst development with a single model, we have chosen instead to focus on a specific question: what is responsible for the high level of organizational robustness observed in the developing mammalian blastocyst. Toward this end, we develop a modeling platform with which different hypotheses can be tested.

To simulate and describe the dynamical and stochastic nature of the developing embryo, we develop a discrete, multiscale and stochastic model that incorporates motions, deformations, and divisions of individual cells as well as gene regulatory processes that are influenced by cell positions and cell-cell interactions. While we do not model the fine grained details of cell structure and mechanics (tight junction formation, cell morphology changes, and cell contractility for example) here, this model accounts for cell-cell interactions, how those interactions drive passive cell motions, and regulatory control over the type and orientation of cell divisions. Therefore, it allows us to test various hypotheses about how these factors may influence embryonic organization. Using this model, we investigate the efficacy of different processes related to the three aforementioned hypotheses at promoting organization. To inform this model and test its predictions, we utilize quantitative immunofluorescence measurements of Oct4 and Cdx2 (Fig 1A) to measure the relative expression levels and follow the cell lineage of potential TE cells using Cdx2-eGFP transgenic mouse embryos.

Results of this investigation show the following. First, while pre-patterning and polarity-based processes have a role in organization, it appears that the inside–outside based mechanism is more likely to be responsible for the high level of robustness observed. Second, gene expression noise at levels measured within developing embryos can enhance robustness of organization by promoting cellular plasticity, allowing the embryo to correct organizational mistakes induced by dynamic cellular motions. Third, asymmetries in those noise sources, predicted by modeling and confirmed with gene expression quantification, provide a mechanism to promote this noise-mediated plasticity without stochasticity overwhelming the systems dynamics. Lastly, mammalian cell fate specification is a gradual, rather than switch-like process, which provides a critical time window for noise to fix inevitable specification errors. Taken together, these results suggest that regulative processes that allow cells to dynamically read their environment and react accordingly have critical roles in organization. Furthermore, while typically noise is seen as a nuisance to robust organization [43–45], some forms of noise can actually aid in correcting errors and establishing proper organization in the system.

## Results

To investigate the mechanism responsible for robust development of the TE / ICM, we construct a spatial model of the evolution of a single cell to a multicellular blastocyst. To gather quantitative imaging data of Oct4 and Cdx2 TFs, embryos were fixed, subjected to immuno-staining, and subsequently compressed to ~1/3 of the embryonic diameter prior to imaging to reduce the impact of fluorescence attenuation (without damaging nuclear morphology). To model this process, we first consider a two-dimensional disc geometry, which both mimics the experimental system and provides a simpler output. Later we relax this assumption and model a spherical, three-dimensional embryo and verify that conclusions from 2D modeling are qualitatively similar to those of 3D modeling. In both cases, the *in silico* embryo is modeled as a collection of discrete cells, each of which can physically deform, move in response to local interactions, undergo division, and change cell type (e.g. ICM / TE) based on time evolving gene expression profiles. See the S1 Text for a description of this physical model, which is implemented using the sub-cellular element method (SSEM) [46–49]. The models discussed in the subsequent sections build upon this core physical model to incorporate different hypothesized mechanisms for cell fate determination and control of organization.

### A stochastic, multiscale model of early mammalian blastocyst development

The canonical Oct4 / Cdx2 transcriptional system (Fig 1B) illustrates how crosstalk between these critical genes lead to cell fate specification [29, 50–55]. We investigate the hypothesis that cell contact mediated positional information affects cell fate specification, potentially by augmenting the Oct4 / Cdx2 bistable network (see Fig 1B). This is based on the observation that Hippo signaling is essential for the regulation of Cdx2 expression [38, 56] and that cell-cell contact activates Hippo signaling, which phosphorylates Yap [57]. This, in turn, regulates Tead4 nuclear localization [35–37], causing a reduction of Cdx2 transcription among inside cells, thus promoting ICM development (Fig 1B). These observations along with others using embryonic stem cell lines [58] are consistent with a regulatory model where the stable states of a bistable gene regulatory system [59] represent TE and ICM lineages respectively [40, 41], and cell-cell contact mediated positional information directs each cell to one or the other lineage.

The precise mechanism that mediates this contact based influence is still the subject of debate, so we do not explicitly include intermediaries. Instead, for simplicity, we assume contact influences Cdx2 transcription directly (Fig 1B). These dynamics are encoded in the following non-dimensional stochastic system

$$\begin{array}{l} \frac{dx_{i}}{dt}=k\left(b+S_{i}+\frac{x_{i}^{n}}{0.5^{n}+x_{i}^{n}}\right)\left((1-I)+I\frac{0.5^{n}}{0.5^{n}+y_{i}^{n}}\right)-x_{i}+\sigma_{Cdx2}x_{i}\cdot \eta \\ \frac{dy_{i}}{dt}=k\left(b+\frac{y_{i}^{n}}{0.5^{n}+y_{i}^{n}}\right)\left((1-I)+I\frac{0.5^{n}}{0.5^{n}+x_{i}^{n}}\right)-y_{i}+\sigma_{Oct4}y_{i}\cdot \eta \end{array}$$ (1)

Here, *x*_*i*_, *y*_*i*_ represent the expression of Cdx2 and Oct4 respectively in cell *i*, the first term in parentheses represents self amplification, the second term in parentheses encodes mutual inhibition, and the final term is a stochastic term intended to mimic the intrinsic (i.e. multiplicative) noise processes in gene regulation [60] with σ representing the strength of those noise sources and η represents a normally distributed white noise term. When σ = 0, the expression is regulated by a deterministic mechanism. In later sections we will consider the effect of asymmetric noise amplitudes *σ*_*Cdx*2_ ≠ *σ*_*Oct*4_, but we begin with the simplifying assumption *σ*_*Cdx*2_ = *σ*_*Oct*4_ ≕ *σ*. See Supplementary Materials (SM) for the fully parameterized system and the scalings that yield this non-dimensional reduction.

This genetic circuit has four important state parameters that modulate its behavior: 1) A non-specific control parameter that determines the total rate of transcription in the system (*k*). 2) The strength of Cdx2 / Oct4 antagonism (*I*). 3) The strength of basal transcriptional rate of individual genes (*b*). 4) A parameter encoding contact mediated asymmetries (*S*_*i*_, positive values indicate outer cell bias, negative indicates inner cell bias). Since this model has a complex parameter space, we first constrain it to a reasonable region using available fluorescence data from [9] and obtain an indicative parameter set that generates ICM and TE expression states consistent with data (See Supporting Information for details). In order to ensure that the model is not being constrained and tested by the “same” data, we choose here to use this pre-existing data to initially constrain the model and subsequently perform new experiments to test its predictions. These constraints are used as an initial guide and we later consider the sensitivity of subsequent results to changes in these critical parameters (*k*, *b*, *I*, *σ*).

### Contact mediated fate specification alone is insufficient to robustly form trophectoderm and inner cell mass structures

It is well established that the mutually inhibitory dynamics between Cdx2 and Oct4 form a bistable system (as indicated by the overlap in Fig 1C) that generates two (or more) distinct cellular states (indicative of ICM or TE in this case). Furthermore, it is shown that by influencing the transcription rate of Cdx2 (increasing or decreasing as proposed in this positional theory) a cell can enter one or the other of those two states (Fig 1C). It is thus clear that the positional mechanism can direct cells to the correct lineage by sensing the cellular environment (Fig 1D, second embryo). Due to cell-cell contact, inner cells inhibit Cdx2 expression, thus promoting Oct4 expression and vice versa for outer cells.

The mechano-chemical transduction of adjacent cell interactions is sufficient to initially pattern the early embryo. However, dynamic whole embryo simulations (Fig 1D, S1 Movie) indicate that unexpected cell motions and divisions, which inevitably occur during normal blastocyst formation, cause intermingling of these cell types, thus destroying this architecture. For simplicity, we initially consider a deterministic version of the regulatory network (thus no stochasticity and setting *σ* = 0), and simulate the full two dimensional model. Results show 1) a number of interior cells mis-expressing TE markers and 2) improper allocation of cells to the ICM / TE lineages. Interestingly, mis-expression is confined to interior cells, with no exterior cells expressing ICM factors (Fig 1D, final snapshot). This is consistent with the findings that Cdx2 expressing cells frequently move from outside to inside [22]. In order to account for simulation stochasticity, we also performed 20 additional simulations (not presented), all showing similar qualitative results.

To understand why mis-expression is confined to interior cells, we analyzed the regulatory network (Eq 1 with *σ* = 0). Fig 1C, which indicates the influence of the contact mediated transcriptional bias *S* on possible states, illustrates the effect of a cell transiting from the exterior to the interior of the embryo (or vice versa). Here, the blue (resp. red) region shows the bias ranges where Cdx2 (resp. Oct4) dominant states are possible. In the overlapping region, the system is bistable and either TE or ICM like states are possible. Upon reducing the bias from the expected outer cell bias to the inner cell bias, the system does not exit the bistable region (note two green dots stay under the blue area). Thus, a change of Cdx2 expression is not forced. On the other hand, in the reverse transition an Oct4-expressing ICM cell that relocates to the exterior (which occurs very infrequently if at all in developing embryos) will exit the bistable region and only the Cdx2 dominated state is possible. This asymmetry in fate switching is a result of contact mediated signaling affecting only Cdx2.

Stronger biasing (decreasing *S*_*i*_) would force expression of ICM factors (and suppression of TE factors). This however requires the differential expression of Oct4 / Cdx2 to be much larger than experimentally observed [9]. We additionally determine the sensitivity of this result to critical model parameters. We compute the critical bias that will drive a TE → ICM transition for a range of values of the remaining parameters (Fig 1E). Results indicate that in a substantial portion of the parameter space (the blue region), no change of bias will force such a transition, suggesting that an additional regulatory mechanism is involved.

To test the potential influence of polarity and the type and angle of division on organization, which have been proposed to positively influence organization, we incorporated (Figure C in S1 Text) observations about cell division [20]. We first incorporated symmetric versus asymmetric divisions into the model. All inner cells were assumed to divide symmetrically to produce two ICM cells. Outer cells were assumed to polarize and divide either symmetrically with a division plane perpendicular to the embryo surface (to produce two TE daughter cells), or asymmetrically with a plane of division parallel to the embryo surface (to produce a TE cell on the outside and an ICM cell on the inside). Results indicate that this mechanism on its own does not lead to accurate organization (Figure C in S1 Text, Model 2), consistent with [42]. It has additionally been observed that the mode of division (symmetric versus asymmetric) of a mother cell and its daughter cells are correlated [20], suggesting a potential compensatory mechanism to ensure proper allocation to ICM and TE lineages. We incorporated this compensatory mechanism along with Bischoff et al.’s data [20] for the frequency of different modes of division at each cleavage stage. Results (Figure C in S1 Text, Model 3) show again that this does not rescue proper organization. Therefore, in subsequent model simulations, we do not prescribe cell division controls—division orientations are random, which is more indicative of the less controlled nature of cell division observed in [61].

We next considered the effect of molecular pre-patterning on organization. It was recently suggested that Sox21 heterogeneity [12] biases cell fate in the early embryo as early as the 4-cell stage. Specifically, they find that the number of cells each 4-cell blastomere contributes to the TE and ICM lineages is correlated with their initial Sox21 expression. While we do not specifically model Sox21 here, we do incorporate this fate biasing to investigate its potential role in organization by assigning individual cells at the 2-cell stage a high or low Sox21 identifier and incorporate data from [12] to bias the type of divisions and lineage allocation of those cells through development. Results (Figure F in S1 Text) show that while this biasing can ensure the proper number of cells is directed to ICM and TE lineages, it does not ensure those cells to be properly organized.

The essential problem with these models is that while they can help initially organize the embryo, they do not have the capacity to correct organizational errors that arise as a result of cell motions, which are observed to occur ([20] and subsequent imaging). Tensile forces and other mechanical factors [61], which are not taken into account here, can further constrain motions and positioning of cells. However while these factors may reduce the occurrence of mis-localized cells, they still would not provide any additional capacity to correct errors once they arise.

### Noise in gene expression promotes cell plasticity and improves organization

How then is proper TE / ICM organization attained in the presence of these cellular motions? Cell type dependent differences in adhesion can sort cells of different identities in a number of settings; this is the so-called Differential Adhesion Hypothesis. However, we note 1) the thermodynamically driven random motions required for this will likely be slow, 2) cell sorting alone cannot control the proper number of cells allocated to the two lineages, 3) evidence supporting such a mechanism is lacking, and 4) cell motions are shown to be unidirectional with outer cells moving to the inside of the embryo only [21]. We thus do not discuss this mechanism further. Alternatively, it has been suggested that in some contexts, noise in gene expression processes can improve cell plasticity, allowing cells to continually read their environment and adjust the fate accordingly [62–65]. We thus next ask what effect intra-cellular noise has on this system.

**Fig 2:**
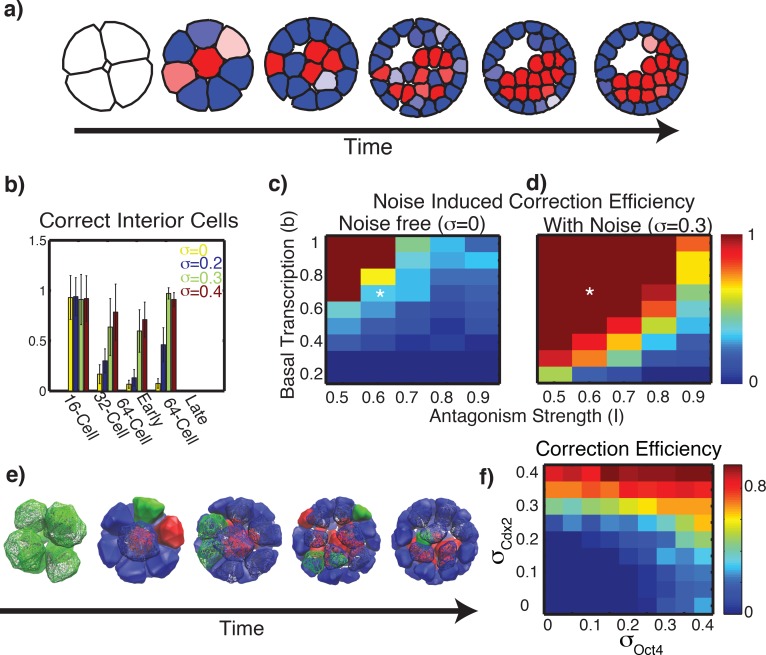
Gene expression noise improves contact mediated fate determination. ***a)*** Sample SSEM simulation, identical to Fig 1D but with stochastic noise included (*σ = 0*.*3*). ***b)*** 3D embryo simulation results showing the fraction of interior cells expressing the correct fate over time. Mean and standard deviation of an ensemble of 20 simulations at each noise level are reported. ***c*,*d)*** Simulated correction efficiency with and without gene expression variation. For each parameter set, relocation of 100 independent TE like cells to the interior is emulated, and the fraction that correct to the Oct4+ state is recorded. Asterisks indicate parameters derived from published data [9], similar to Fig 1E. ***e)*** Sample 3D simulation with σ *= 0*.*3*. ***f)*** Dependence of plasticity on noise asymmetry. One hundred independent cells are simulated for different combinations of (σ_*Cdx2*_, *σ*_*Oct4*_). Each cell is initialized in a TE state, the bias *S*_*i*_ is adjusted from 0.6 → -0.2, and the fraction of cells that transition to an ICM state by T = 48 is recorded. Unless otherwise stated, the base parameter values for all simulations are the same as for Fig 1D.

We introduced stochasticity (σ>0) into the gene regulatory system and performed an identical simulation to that in Fig 1D (all other model specifics were held fixed and only σ changed). Results (Fig 2A) indicate that the addition of stochasticity is sufficient to generate an accurately patterned embryo. This simulation experiment was repeated with a more representative 3D geometry (holding all aspects of gene regulation the same as in Fig 2A) with results (Fig 2E) again indicating that this cell-contact based mechanism in combination with noisy gene regulation is sufficient to accurately organize the embryo. Additional simulations with the same model parameters that were performed to account for simulation stochasticity showed similar results.

This suggests noise induced plasticity has the capacity to improve organization. We next ask whether such effects may actually be present in the embryo. Since stochasticity cannot be easily “perturbed” to assess its influence, we ask whether the amount of expression noise within embryos is consistent with the amounts required to provide a benefit. First, to determine the influence of the amount of gene regulatory noise in the system, we performed the same numerical experiment with the 3D model and varied the strength of stochasticity (σ). For each noise level, 20 independent simulations were performed and the accuracy of organization was assessed at different time points (Fig 2B). As with Fig 1D, we find that in all cases mis-expression is almost exclusively limited to inner cells. Thus, to quantify the accuracy of blastocyst organization, we determined the fraction of interior cells that were Oct4 dominant (e.g. high in Oct4, low in Cdx2) at the blastocyst stage. Results indicate a clear positive effect of this noise on the final organization (Fig 2B, S2 Movie), thus noise leads to a substantial reduction in the number of mis-expressed cells. Additionally, it appears that there is an optimal noise level σ = 0.3, above which no additional benefit is found. Indeed, above this level excessive noise begins to drive erroneous transitions in the wrong direction and begins to overwhelm the dynamics of the system.

Second, to consider the sensitivity of these results to parameter variations, we perform a simulation study of Eq 1 with partially randomized parameters, for both the noise free and stochastic systems (with *σ*_*Cdx*2_ = *σ*_*Oct*4_). The parameters *b* and *I* are taken to be the same for all cells, independent of location and time. We grid these parameters and for each set, simulate 100 independent cells. Each is initialized at a TE state with a randomized combination of *k* and *S*_*i*_ *>0* (constrained to parameters that give rise to distinct TE states). Subsequently, each cell is re-assigned a randomized value of *S*_*i*_ *<0* (to mimic relocation from outside to inside), simulated in time, and the fraction that correct to the interior lineage is recorded (Fig 2C and 2D). Results show that noise substantially enlarges the region where the state correction occurs robustly. Note that the stared parameter values represent those determined by constraining the model with the published data by Dietrich et al. [9]. These values were constrained solely by this prior data independent of this and all subsequent analysis of the model. Thus, noise expands the effective operating regime to include the neighborhood of this parameter set.

We make a brief note about the use of expression data [9] to constrain the parameters in Eq 1. Fluorescence intensity ratios were used to measure the difference between Cdx2 and Oct4 expressing cell types. While a number of experimental factors such as antibody affinity can potentially influence this estimate, we expect the actual expression differences to be larger than the assumed ranges. Increased expression differences would arise from increased strength of antagonism (increased *I*), decreased basal transcription rates (decreased *b*), or both (see Figure A in S1 Text). Either of these would shift cells to a state where noise becomes increasingly necessary for proper function (Fig 2C and 2D). Thus, if the relative expression estimates derived from data [9] are strongly influenced by experimental methods, we expect those estimates to be conservative, thus leading to underestimation of the importance and need for stochasticity in maintaining plasticity. Combined, these results support the hypothesis that gene expression noise has a substantial positive influence on TE / ICM organization and adds significant robustness for the correct structural arrangement of mouse blastocysts.

We next ask if noise in Cdx2, Oct4, or both appear to be driving the cell fate transitions required to correct mis-expressions and improve organization. Our 2D and 3D simulation studies thus far have indicated there is an inherent asymmetry in this system. Specifically, cell fate errors are almost exclusively localized to the interior of the embryo, e.g. Cdx2 positive cells are found in the inside. This is consistent with previous observations [20, 21] that cells translocate from the exterior to interior of the embryo. Thus, noise must (almost) exclusively drive transitions from the TE lineage to the ICM lineage. We consider the possibility that noise in one factor may be more influential than noise in the other. To test this, we allowed noise levels (σ_Cdx2_ and σ_Oct4_) in the gene regulatory system (Eq 1) to be different among factors. We fixed all kinetic parameters at their base values and performed a simulation experiment similar to Fig 2C and **2D**. For an array of values of the two noise strengths, a cohort of *in silico* cells were initialized in a TE like state, the bias (*S*_*i*_) was changed to reflect a position in the interior of the embryo, and the fraction that change fate is recorded as a measure of the effectiveness at promoting transitions (Fig 2F). Results show noise in Cdx2 is more effective at driving the TE → ICM transition than noise in Oct4, which has almost no influence.

Combined, these results provide two predictions that we test next: 1) there is an optimal band of noise levels (around σ~0.3) that are sufficiently large to provide a benefit, yet sufficiently small to avoid overwhelming the system, and 2) higher Cdx2 variability combined with reduced Oct4 variability would be optimal in the sense that sources of noise that are useful reside in this range while sources of noise that are not of value are reduced.

### Quantification of gene expression noise

**Fig 3:**
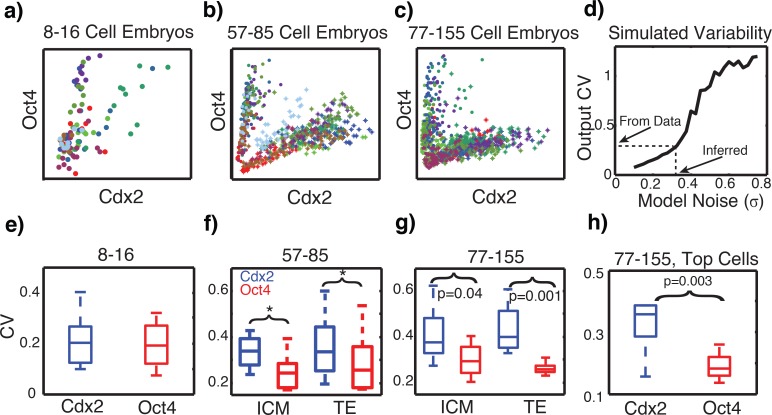
Quantifying intra-embryonic variability of Cdx2 and Oct4. ***a-c)*** Expression as a function of cell stage for 8–16 (16 embryos, mean cell number = 10.3), 57–85 cell embryos (10 embryos, mean cell number = 76), and 77–155 (8 embryos, mean cell number = 115). Each dot represents expression of a single cell and each color identifies cell from a single embryo (all red points are cell in the same embryo for example). These results are comparable to those in the fourth figure of [9]. ***d)*** Variability of simulated populations for different values of σ. For each, 100 cells are simulated and the CV is computed at T = 48. The output value (0.3) indicated is a rough measurement from panels f,g. Representative ICM state parameters *S*_*i*_ = *-0*.*2*, *k = 0*.*7* are used. ***e-g)*** Variability of Oct4 / Cdx2 from the cohort of embryos in panels a-c. The coefficient of variation (CV) for the ICM (red) and TE (blue) subpopulations of each embryo is computed. Box plots indicate the variation of this noise measure across embryos. Stars indicate significance of the indicated at p = 0.01 (paired t-test). Prior to the ~16 cell stage, the ICM and TE have not formed, so noise is quantified treating all cells in the embryo as a single population. ***h)*** Variation of expression among only TE cells at the top of embryos reported in panel *g*.

To test these predictions, the levels of expression variability of Cdx2 and Oct4 TFs in mouse embryos were quantified. Quantitative immunofluorescence staining of mouse embryos at different developmental stages was performed using Cdx2 and Oct4 antibodies (Fig 3). To eliminate variability among experimental treatments, embryos at different stages were fixed, stained, and co-imaged at the same time. For each embryo imaged, the nuclear expression of Cdx2 and Oct4 was quantified for individual cells using a 3D segmentation pipeline (Fig 3A–3C). Independent data sets were obtained from 34 embryos. The coefficient of variation (CV) of each of these data sets was then determined, giving four intra-embryonic noise measurements of these embryos. At the earliest stage (8–16 cells), gene expression changes have not yet begun and cells cannot be classified as interior or exterior, thus for this stage we treated all cells within the embryo as a single population rather than distinguishing between TE and ICM. These noise quantifications (Fig 3E–3H) indicate that at 57–85 and 77–175 cell stages, the measured noise levels are in the 20–40% range. Some of this variability is inevitably measurement noise and as such this should be viewed as an upper bound for the intrinsic variability.

To compare these measured noise levels to those used in previous simulation studies (Fig 2), we numerically determine the relationship between noise amplitude (σ in the model) and resulting level of gene expression heterogeneity (which is measured in Fig 3). One hundred independent stochastic simulations were performed at each of a range of noise inputs (σ), the CV of the resulting population was recorded at each, and the input / output relation (Fig 3D) was used to determine this relationship. Interestingly, noise input and output variability are roughly linearly related below *σ* = 0.3, the rough level inferred from data. Further, a dramatic change in behavior occurs above roughly *σ* = 0.4, where noise was found to start to overwhelm the system in simulation studies, causing random transitions between ICM and TE states (Fig 2B). These results suggest that not only may stochastic effects improve organization from a theoretical perspective, but that levels of variability observed within embryos fall into the range predicted to provide this benefit.

Noise quantification results (Fig 3F) additionally show the CV for Cdx2 (~35%) is significantly larger than that of Oct4 (~25%). This asymmetry was confirmed by an independent experiment. Importantly, we note that Cdx2 and Oct4 noise asymmetry is not present at the 8–16 cell stage, but occurs at subsequent stages and is maintained even in 100+ cell embryos. Several pieces of evidence suggest that the observed noise asymmetry is not an artifact. First, to ensure these results are not an artifact of image attenuation at different focal depths, we measured variation in only TE cells located in the top layer of the embryo (Fig 3H). Second, we reduced the nuclear mask size during segmentation, and found the same results, indicating that this is not due to segmentation artifact (Figure I in S1 Text). Third, we considered different methods of normalizing the fluorescence intensity data (by DNA content, cell volume, or no normalization, Figure K in S1 Text), and found qualitatively the same results. Fourth, we analyzed previously published qPCR data at the 32-cell stage [66] and a similar asymmetric transcriptional noise variation was detected for Cdx2 and Oct4 (Figure J in S1 Text). Lastly, we swapped secondary conjugates and saw the same results (Figure K in S1 Text). These results suggest that the observed noise asymmetry did not arise from experimental artifacts. While we focus on Cdx2 and Oct4 expression here, it was previously observed [9] that Nanog also exhibits significant levels of heterogeneity. This further suggests that the asymmetry we observe between Cdx2 and Oct4 is not a general asymmetry between all TE / ICM associated factors but rather may be specific to those two.

These results are consistent with the modeling prediction that noise in Cdx2 expression would improve organization while noise in Oct4 expression would not. Taken together with the predictions of the modeling results, we conclude that noise is in the appropriate range and has the appropriate structure to help promote proper organization of the embryo.

### Evidence of cell plasticity and importance of timing in fate decision

**Fig 4:**
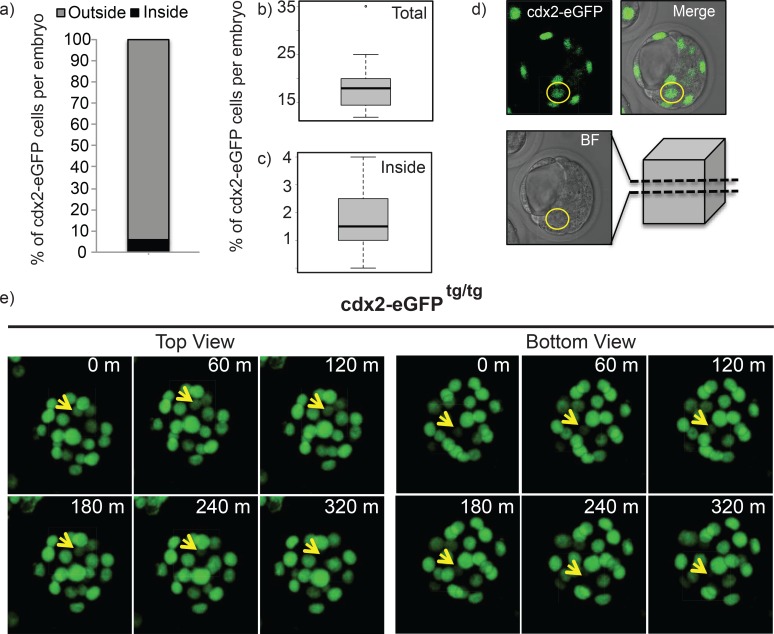
Cdx2-eGFP expression in inner cells of embryos between late morula and early blastocysts. a) Percentage bar graph showing the location of Cdx2-eGFP positive cells in blastocysts. b, c) Whisker box plots showing the total number of Cdx2-eGFP positive cells per embryo and the number of Cdx2-eGFP positive cells found in the inner portion of embryos. d) a single slice representation of an early blastocyst embryo showing a Cdx2-eGFP cell located inside (yellow circle). e) Top and bottom view time frames of 3D reconstruction of an embryo transitioning to an early blastocyst stage. Yellow arrows indicate a cell that expresses eGFP transiently. m: minutes, BF: bright field.

The results shown above indicate that noise-mediated cell plasticity has a role in correcting expression errors that arise when cell divisions and motions cause intermingling of different cell types. To determine if such intermingling occurs, we performed live imaging of Cdx2-eGFP transgenic mouse embryos, in which a fusion protein construct was knocked into the *cdx2* locus [22]. The half-life of eGFP in this context is expected to mimic that of Cdx2. We performed 6 independent time lapse imaging experiments starting with morula stage embryos, of which a majority developed to normal blastocysts, as examined by external morphology (96%, n = 54 out of 56, S4 Movie). Approximately 9% of Cdx2-eGFP positive cells initially located at the outer layer of blastocysts (41 out of 437 cells, n = 24 embryos) relocate to the ICM (Fig 4). This represents approximately 5% of embryonic cell populations that will mis-express Cdx2 in the ICM. This is, in good agreement with observations made in [20, 21].

At present, we are unable to determine the cell fate of the Cdx2-eGFP positive cells relocated into the ICM at the blastocyst stage. However, evidence suggests that the inner cells expressing Cdx2-eGFP adopt the new ICM fate. First, during 12–18 hours of continuous live imaging, none of Cdx2-eGFP expression detected in ICM moved back to the outer layer of blastocyst (Fig 4E, and S3 Movie). Second, Cdx2-eGFP expression decreases once outside cells move inside (S3 Movie and [22]), suggesting that these cells lose TE cell identify. Third, when Watanabe et al. [21] examined the movements of individual cells, movement of inside cells to outside was never observed. These findings are consistent with the view that the Cdx2-eGFP positive cells inside remain inside and dynamically readjust their fate based on position.

A key requirement of a position-based mechanism is the presence of positional information that the cell can sense and transduce the information. This raises two interesting issues. First, how does the embryo cope with the initial ambiguity when fewer than 8 cells are present as all blastomeres are exposed to the zona pellucida (i.e. there are no interior cells)? Second, how does the embryo cope with the lack of E-cadherin expression prior to compaction [67], which is suggested to be the first step in this cell contact mediated signaling cascade [36]? When E-cadherin is inhibited (using E-cadherin blocking antibody ECCD1) in 8–16 cell compacted embryos, further development ensues but ICM cells are either absent or significantly reduced [36, 68]. Consistent with this observation, Yap localization is indistinguishable between interior and exterior cells of these embryos [36]. Thus, current evidence suggests that prior to compaction, contact-mediated positional information is both ambiguous and cannot be transduced.

**Fig 5:**
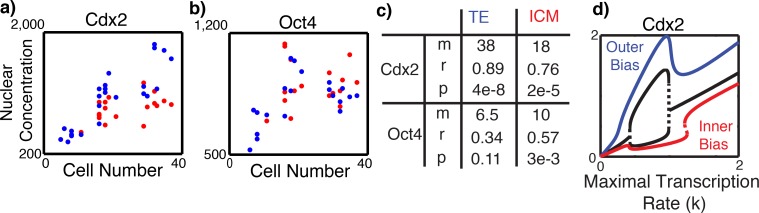
Transcriptional suppression slows down fate decisions until sufficient spatial information is present. ***a*,*b)*** Intensity of Cdx2 and Oct4 respectively versus “embryo age” as measured by cell number. Each data point represents the mean normalized intensity of in *ICM* (red) or *TE* (blue) cells of a single embryo (24 embryos with mean cell number 20). ***c)*** Statistics for data in panels *a*,*b*. Slopes of the best-fit regression line (m), correlation between cell number and expression (r), and significance of the correspondence between intensity and cell number (p, paired t-test) are provided. ***d)*** Dependence of Cdx2 expression on *k*. Blue (*S*_*i*_ *= 0*.*6*), red (*S*_*i*_ *= -0*.*2*), and black (*S*_*i*_ *= 0*) curves represent indicate different (un)biased settings. In this bifurcation diagram, solid (dashed) lines indicate stable (unstable) steady states. Remaining parameters are *n = 4*, *I = 0*.*6*, *b = 0*.*7*.

The natural solution to the problem is that cells can defer fate specification until after compaction. To determine how Cdx2 and Oct4 gene expression changes with time, we performed a pseudo-longitudinal analysis of Cdx2 / Oct4 expression. A cohort of embryos were fixed, grouped together, and subjected to immuno-staining within the same reaction mixture. Cells were visually classified as either interior or exterior and expression as a function of cell number (a surrogate for time) was recorded (Fig 5A and 5B). Fig 5A shows both populations begin with similar initial Cdx2 expression, which increases in both cell populations, but more markedly in exterior cells (replication confirmed this trend, Figure H in S1 Text). The dynamics of Oct4 expression are less clear (Fig 5B), however expression increases are larger, more highly correlated, and more significant in the ICM than in TE cells (Fig 5C). We thus suggest that 1) fate commitment is a gradual (takes multiple hours and days, consistent with [14]) rather than a quick, switch like process as it is often portrayed and 2) as time progresses, positional information becomes more robust in the sense that when more cells are present, the distinction between “inside” and “outside” is more clear. Combined, these indicate that the timing of commitment may aid organization by ensuring that commitment does not occur prior to reliable information becoming available.

Presently, it is unclear what is responsible for either the delayed or gradual nature of commitment. Based on the regulatory motif in Fig 1B, we raise one potential candidate. A gradual increase in the non-specific (in the sense that it targets all cells and transcription factors equally) rate of transcription (*k* in the Eq 1) would lead to gradual commitment (Fig 5D). At low rates, the system is monostable (Fig 5D, black curve), while at higher rates bistability arises (cells are bipotential) and the proposed Cdx2 transcriptional bias would direct cells to follow a different paths (blue versus red in Fig 5D).

This is by no means the only potential candidate. It is however consistent with the fact that gene expression divergence occurs through the preferential increase of one transcription factor rather than decrease in the other (which would occur if for example inhibition changes over time). Furthermore, an increase in *k* over time could result from the maternal to zygotic transition. While we have suggested transcription / translation control is responsible for this delay, modulation of transcription factor degradation via proteasome activity (for example), would provide a similar outcome as well.

### Summary of the underlying mechanism and the influence of stochasticity

**Fig 6:**
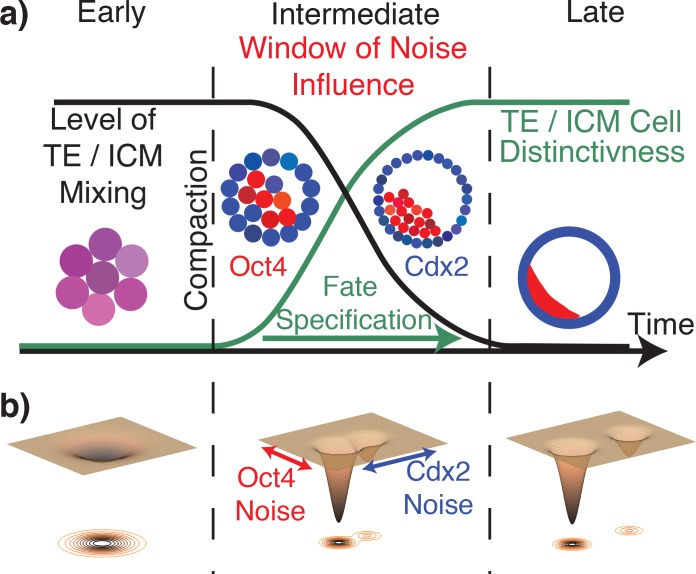
Schematic of the gradual process of trophoectoderm (TE) and inner cell mass (ICM) formation. ***a****)* Schematic of the distinctiveness of TE / ICM cells and the mixing of the two populations as time progresses. ***b****)* Schematic of the hypothesized epigenetic landscape as a function of time. Prior to compaction, the system is mono stable and all cells are equivalent up to stochastic fluctuations. After compaction, this state bifurcates to two possible states, with the relative stability of the two determined by local cell-cell communication. Arrows schematically indicate how different sources of noise could be (non)constructive by driving fluctuations in different directions in the underlying landscape. At later stages, the ICM / TE gene expressions gradually diverge leading to more distinct states that are more difficult to transition between.

The distinctiveness of TE / ICM cells and the mixing of the two populations is schematically depicted in Fig 6A. Shortly after compaction (~16 cell stage), all cells undergo the gradual process of distinguishing themselves through changes in gene expression. This creates a window of time in which noise has a critical positive effect on organization, namely by correcting mis-expressions that arise as cells move and divide. We hypothesize that the stochastic effects studied here are intimately related to the structure of the so-called genetic landscape of the circuit regulating cell fate [69, 70]. This view proposes that gene expression is a manifestation of minima in an underlying energy landscape and mis-expressing cells are stuck in shallow, less favorable minima (Fig 6B). Noise then drives state transitions by pushing that cell over a saddle. From this point of view, our results indicate that the levels of stochasticity we quantified (Fig 3F and 3G) in embryos may be both beneficial to organization and near some form of optimum (i.e. both sufficiently high to improve organization but still sufficiently low to avoid overwhelming the system). The asymmetries of cell fate corrections in simulation studies along with the quantified asymmetry of expression variability further suggest that not all noise is created equal. In particular noise in Cdx2 appears to be the primary driver of cell fate correction while noise in Oct4 provides no additional benefit.

In response to this idea it is natural to ask, if noise has the propensity to drive beneficial transitions, why would it not undo those transitions? This is because state transitions are not symmetric. While a system may have multiple stable attractor states, the relative stability of those states need not be the same. In particular, Fig 6B depicts a scenario where the “incorrect” cell state is less stable (i.e. a shallower trough) than the “correct” cell state. In this scenario, the noise required to drive a correct → incorrect state transition is significantly larger than that required to drive the reverse transition. Thus, once noise has driven a cell to the correct state (i.e., more stable state), it would become locked (unless position again changed).

This analogy also presents an interesting interpretation of the gradual process of fate commitment (Fig 5). In the landscape view, gradual commitment would correspond to a steady divergence of the representative attracting states (depicted in Fig 6B). As cell fates become more distinct, it becomes more difficult to stochastically drive transitions between them. Thus, gradual commitment would endow the system with a temporally varying level of plasticity. A consequence of this would be that in the earlier, more dynamic stages of development, cell fates would be more fluid and the system could readily correct errors. In later stages (once ICM / TE structures have largely formed), cell fates would become more distinct, with a commensurate reduction in plasticity. In other words, the slow nature of this process would create a time window where noise drives correction of inevitable specification errors.

### Alternative mechanisms and model limitations

While contact mediated biasing of Cdx2 transcription, along with stochastic effects, would be sufficient to ensure accurate organization of the early embryo, it is of course not the only potential mechanism. Recent observations have shown that DNA binding properties of critical transcription factors correlate with cell position [13]. While a full analysis of this hypothesis at the DNA binding level will be left for future work, we briefly consider the possible effects of this. In particular, Cdx2 has longer residence times in outer cells. This could positionally affect, among other things, Cdx2 transcription (if Cdx2 auto-amplifies its own transcription) or degradation rates of Cdx2 (if for example, DNA binding prevents post-translational modifications leading to degradation). The former would lead to outer cells exhibiting larger Cdx2 transcription, which is accounted for in the current model, and thus would have a similar effect. We tested the latter possibility by assigning higher (resp. lower) degradation rates to inner (resp. outer) cells. Results (Section 3.2 in S1 Text and Figure D in S1 Text) show that without any contact mediated transcriptional biasing (*S = 0* for all cells), positional dependent Cdx2 degradation rates promote accurate organization. When this mechanism and the previously discussed cell-cell contact mediated biasing of Cdx2 transcription are both included in the model, they combine to organize the embryo, indicating they do not interfere with each other and could potentially act as parallel pathways to promote organization. A caveat of these results is that in both cases, stochastic effects are still required to ensure organization.

An alternative hypothesis is that cell polarity influences Cdx2 transcription [71]. The basis of this model is that Hippo signaling is suppressed by the apical domain in polarized outer cells [38, 72]. In comparison, the model discussed above is based on observations that cell-cell contact promotes Hippo signaling in interior cells. Functionally, both pathways would tend to promote Cdx2 transcription in outer cells and inhibit it in inner cells, both by modulating Hippo signaling. While these two mechanisms are molecularly distinct, at the scale of the modeling performed here, they would both enter into the model as a positional bias of Cdx2 transcription, but with a different interpretation of the positional dependent parameter (*S*). Hence, they would lead to the same predictions, namely that apical domain control over Cdx2 transcription would provide the same capacity to correct specification errors and promote organization.

Mammalian blastocyst development has generated sustained interest for decades that has only accelerated in recent years. Those investigations have yielded a wealth of observations leading to numerous proposed models and molecular mechanisms promoting organization. We emphasize that the model presented here does not account for all aspects of development or match all observations about mammalian blastocyst development. As such, we do not include the molecular details of transcription, translation, and protein stability and will not make statements as to the influence of factors such as Nanog or Sox2. Fluid transport and physical factors, such as cortical tension, filopodia dependent processes [73], or cell contractility [74], which can affect motions of cells, are not included in the current model. Here our goal is not to suggest “the model” of development. Rather, our model is intended to shed light onto the question, what contributes to the high level of robustness observed in early mammalian development.

## Discussion

An important challenge in developmental biology is to understand the design principles responsible for spatio-temporal organization of the embryo. While a number of mechanisms have been proposed to contribute to this process [3, 4, 20, 30, 31], a positional based regulative model has received significant recent support. The central premise of this model is that cells communicate directly through cell-cell contact via Hippo signaling to affect downstream expression of the critical cell fate regulators Cdx2 and Oct4 [35–37]. Consequently, a cell’s position determines its fate. This model is attractive because it has the potential to add a level of robustness to previously proposed theories. Since positional information is a dynamic quantity that can change in time, cells may continuously read their environment and adjust the fate accordingly. Here, we combine modeling and quantitative image analysis to show that our model can ensure the robust development of normal blastocysts, provided stochastic effects are considered.

It is clear that in a perfect world, cell contact signaling is sufficient to direct organization. However, the early embryo is in a dynamic environment with cells changing position [20, 21]. While previous work suggests that cell polarity and regulation of the orientation of cell divisions aids organization [20, 32], live imaging of Cdx2-eGFP transgenic mouse embryos (Fig 4) and tracking of cell divisions [21] indicates that this alone is insufficient ensure all cells remain in the appropriate location within the embryo. Thus, some mechanism to correct mis-localized cell fates within an embryo and ensure the limited number of mammalian embryos undergo successful preimplantation development is required. How does the embryo cope with these out of place cells? Adhesion mediated sorting has been suggested in many settings to direct tissue segregation [42]. The absence of movement of cells from the inside of the embryo to the outside [21], however, argues against this since mis-expression is found primarily in the interior. Furthermore, it provides no way to balance the number of cells that commit to the ICM and TE lineages. The regulative positional based mechanism provides a natural framework to account for this, namely that cells simply change their fate upon relocation. While this is intuitively plausible, our modeling results (Fig 1C–1E) show that changes in local signaling information (upon relocation), on their own, are insufficient to guarantee fate corrections in many cases.

*In silico* modeling and quantitative imaging results here suggest that stochasticity in gene expression processes is sufficient to improve plasticity and to ensure accurate formation of TE / ICM structures. While we cannot directly probe the influence of stochasticity by selective perturbation, our modeling predicts the magnitude of noise sources and provides an experimentally supported hypothesis for what drives cell fate corrections. This raises an important point that while historically, noise has been seen as a nuisance threatening organization [75, 76], more recent work has begun to recognize its potential benefits [44, 64, 65]. Results here are in line with this theory. First, they indicate that the system may have found a way to optimize the amount of gene expression noise present so as to provide the maximum benefit of noise-mediated plasticity without overwhelming the systems dynamics with excess noise. Second, noise quantification and simulation results suggest that the structural asymmetry in the noise may further aid organization. Finally, the gradual nature of commitment could provide a means for stochastic effects to influence organization during the early, more dynamic phase of organization, while reducing the effects of stochasticity at later stages, once the TE / ICM structures have already formed. Intuitively, stochasticity has the role of preventing the embryo from getting stuck in an undesirable meta-stable state. In particular, noise may introduce perturbations, leading to switch of cell fate from an undesirable state, which is less stable, to a desirable state that is usually more stable. Thus, while the regulative model is a plausible and even compelling mechanism to organize the embryo, these results indicate that noise has a critical role in helping this mechanism deal with the ambiguities and imperfections of positional information that are central to it.

Given the ubiquitous presence of many of the elements of this system in other contexts, we expect observations regarding the positive influence of noise on organization to have broader biological implications. Bipotent gene expression circuits are used in a wide variety of biological systems ranging from embryonic development [77], to hematopoietic [69, 70] stem cell differentiation, to osteo-adipo progenitor dynamics [78]. For example, later in embryonic development ICM cells are further organized into the epiblast (the future embryo) and parietal endoderm (the future visceral endoderm). While local cell-cell communications (e.g. through direct physical or biochemical signaling) or long-range patterning cues (e.g. morphogens) may initiate desirable spatial organizations, we hypothesize that the stochastic nature of those processes requires additional mechanisms to ensure precision. It will be important to determine whether gene expression noise and their asymmetries are more generally incorporated into various decision-making processes in development such as cell-cell boundary formation during organogenesis and the emergence of tumors.

Our findings do raise a number of important questions. First, what might throttle transcription (or related processes) prior to compaction and is that control coincident with or directly tied to the occurrence of compaction? While we have not identified a responsible regulator, we expect it to act globally, affecting all cells commensurately. Second, how are noise levels controlled or “tuned”? Numerous theories have been proposed for noise attenuation, but how would it be “controlled” rather than simply “attenuated”? One hypothesis is that feedback loops with other factors influence stochasticity. For example, among embryonic stem cells, expression variability of genes are regulated through Nanog dependent feedbacks [79]. Lastly, how are noise levels in different factors modulated differently? One possibility is to simply modulate mRNA copy number, which influences variability [43]. Alternatively, transcription factor / promoter binding dynamics may provide a mechanism to tune transcriptional bursting frequency [80], leading to “tunable” variation. Regardless of the source of this control, these results suggest noise promotes cellular plasticity and has an important positive influence on blastocyst organization.

## Materials and Methods

### Ethics statement

Our Animal study was approved and carried out by following Institutional Animal Care and Use committee (IACUC) guideless at the University of California, Irvine (protocol number 2008–2814). Females were euthanized with CO_2_ and cervical dislocation, accordingly to University Laboratory Animal Resources (ULAR) standards.

### Simulation methods

See S1 Text for computational methods.

### Embryo acquisition

Preimplantation stage mouse embryos were collected from 3-week old females (CD1, Charles Rivers and Cdx2-eGFP, Jackson Laboratories). Embryos were obtained after super ovulation with pregnant mare serum (PMSG) and human chorionic gonadotropin (hCG) at desired embryonic day (E2.5, E3.5, and E4.5) by flushing oviducts and uterine horns with holding media (DMEM containing Hepes). Cdx2-eGFP preimplantation mouse embryos were isolated at e2.5 and set for culture in 25μl KSOMaa. Embryos were kept at 37°C under 5% CO_2_ before imaging.

### Live imaging

Transgenic mouse embryos harboring Cdx2-eGFP [22] were imaged using Zeiss 780 confocal microscope at 63x using a 1.4 NA objective. Z-stacks of 2μm slices were collected every 15 minutes with 488nm wavelength laser power (5%), 1024X1024 pixels. Embryos were imaged in 25μl microdroplets of KSOMaa covered with mineral oil in a incubation chamber kept at 5% CO_2_ and 37°C.

### Immunofluorescence

Mouse embryos were rinsed in Acid Tyrode’s solution, (Sigma Aldrich) and fixed in 3.7% formaldehyde on ice for 30 minutes. Primary antibodies used were anti-Oct4 (1:250, Santa Cruz Biotechnology) and anti-Cdx2 (1:200, BioGenex) and secondaries were Alexa555 and CruzFlur647 (see Table D in S1 Text). DNA was stained with Hoechst (2μg/ml, Sigma Aldrich). Embryos were placed on a glass slide coated with a 1% agarose pad and compressed to a 3:1 aspect ratio. All confocal images were acquired using a 63x 1.4 NA objective, on an Axioobserver Z1 Zeiss 780 confocal microscope with Zen2009 software. Z-stack images were acquired at 0.3μm intervals.

### Image analysis

We used custom software to quantify average fluorescent intensity in individual nuclei. See Supplementary Material for details. For live embryo imaging analysis and 3D reconstructions Fiji-imageJ was used.

## Supporting Information

S1 Text This file contains all supplementary text describing computational methods and image analysis. It further contains all supplementary tables and figures referenced herein.
(PDF)

S1 Movie Model simulation. Movie depicting a 2D simulation of the developing blastocyst without gene expression stochasticity.
(M4V)

S2 Movie Model simulation. Movie depicting a 2D simulation of the developing blastocyst with gene expression stochasticity.
(M4V)

S3 Movie Live imaging of an early Cdx2-eGFP mouse blastocyst. A Cdx2-eGFP+ cell moves from the outside to the inside of the embryo while decreasing the Cdx2 expression.
(AVI)

S4 Movie Time lapse imaging of Cdx-eGFP mouse embryos. Transmission video of Cdx2-eGFP embryos demonstrates normal developmental morphology under our imaging and culturing conditions.
(AVI)
