# Supplementary material for: Gene Expression Noise Enhances Robust Organization of the Early Mammalian Blastocyst
Source: PLoS Comput Biol. 2017 Jan 23;13(1):e1005320. doi: 10.1371/journal.pcbi.1005320 (PMC5293272; doi:10.1371/journal.pcbi.1005320)
Supplement: S1 Text — It further contains all supplementary tables and figures referenced herein. (PDF) [file pcbi.1005320.s001.pdf]

Supplemental Material for
Gene expression noise enhances robust organization of the early mammalian blastocyst

William R. Holmes[1*], Nabora Soledad Reyes de Mochel[2,4*], Qixuan Wang[2,3], Huijing Du[2,3], Tao Peng[2,3], Michael Chiang[2,4], Olivier Cinquin[2,4], Ken W.Y. Cho[2,4,**], Qing Nie[2,3,**]

[1] Department of Physics and Astronomy, Vanderbilt University, Nashville TN
[2] Center for Complex Biological Systems
[3] Department of Mathematics
[4] Department of Developmental and Cell Biology, University of California, Irvine CA
[*]These authors contributed equally
[**]Co-corresponding author (qnie@math.uci.edu, kwcho@uci.edu)

1 – Extended Experimental Methods.

1.1 – Image Analysis

We generated segmentation masks for individual nuclei in fluorescence microscopy images of the early mouse embryo. First, we manually annotated cell centers based on DNA images (Hoechst stain). Cell labels (such as inside/outside/ICM/trophectoderm) were manually assigned at this stage. Second, we preprocessed the DNA image for segmentation by normalizing, blurring, and inverting the image. Third, we generated segmentation masks by running active contours initialized from the annotated cell centers (De Solorzano et al., 2001). Expansion of active contour boundaries was constrained by areas of low intensity in the original DNA image and by collisions with neighbors. Lastly, we quantified Oct4/Cdx2 concentration by computing the average pixel intensity in individual nuclear segmentation masks.

Embryos in each experiment were imaged in the same session; this enabled direct comparison of fluorescence contents across embryos in the same experiment. In order to minimize the impact of fluorescence attenuation along the z-axis of the image, in Figure 3h we only used "top layer" nuclei in subsequent analysis (nuclei whose projection to the top of the stack was at least 10% free of overlap with nuclei closer to the top of the stack): fluorescence from “top layer” nuclei is minimally attenuated since there is a minimal amount of tissue to traverse. In addition, mitotic and polar body cells were annotated based on DNA morphology and excluded from subsequent analysis. Image visualization was performed using ImageJ (NIH http://imagej.nih.gov/ij/).

In order to test the robustness of our results against different segmentation parameters, we generated smaller segmentation masks that did not cover the entire cell nuclear region by adjusting active contour parameters. Our results remain qualitatively unchanged, and thus our results are robust against different segmentation parameters.

1.2 – Computational Methods

All bifurcation analysis was performed using MatCont (Dhooge et al., 2003), a MatLab based pseudo arclength continuation package. Spatial model simulations were performed using the sub-cellular element method (Christley et al., 2010; Newman, 2005, 2008; Newman and Grima, 2004). All non-spatial model simulations were carried out with a custom Euler-Maruyama scheme in MatLab (MathWorks). Simulation codes can be found at *http://cmcb.math.uci.edu/software.html*. All visual representations of 2D and 3D simulation results are surfaces extrapolated from the location of the internal elements comprising each cell. For 3D visualizations, these representations were rendered using Visual Molecular Dynamics (VMD) (Humphrey et al., 1996).

2 – Extended Model Description and Analysis

2.1 - Gene Regulatory Network Model Reduction

We consider the following representation of the Oct4 / Cdx2 transcriptional system

$$\begin{aligned} x' &= \left(b_x + a\frac{x^n}{\theta^n + x^n} \right)\left((1-I) + I\frac{\theta^n}{\theta^n + y^n} \right) - \delta x, \\ y' &= \left(b_y + a\frac{y^n}{\theta^n + y^n} \right)\left((1-I) + I\frac{\theta^n}{\theta^n + x^n} \right) - \delta y \end{aligned} \tag{0.1}$$

where *x,y* represent the expression of Cdx2 and Oct4 respectively. For the moment we neglect the stochasticity, since it does not affect the reduction process. The parameter definitions are as follows: $b_{x,y}$ represent the basal rate of transcription within an individual cell. a represents the maximal contribution of autocatalysis to the net rate of transcription. n is a Hill coefficient governing both the strength of autocatalysis and inhibition. Different Hill coefficients are possible for auto-activation and inhibition functions, but we assume $n=4$ for each for simplicity. θ is the EC50 of the respective non-linear interactions, which we assume to be the same for each Hill function. I represents the strength of the mutual antagonism, which assumed symmetric. δ is the rate of degradation, again assumed the same for Oct4 and Cdx2. We have thus assumed that the transcriptional network is symmetric except for differences in the rate of basal transcription, which is where positional differences are assumed to influence the network.

We non-dimensionalize this system by setting $\tilde{t} = \delta t, \tilde{x} = x/(2\theta), \tilde{y} = y/(2\theta)$. Inserting these into Eqs. (0.1), and dropping the tilde, this system reduces to

$$\begin{aligned} x' &= k\left(b + S + \frac{x^n}{0.5^n + x^n} \right)\left((1-I) + I\frac{0.5^n}{0.5^n + y^n} \right) - x, \\ y' &= k\left(b + \frac{y^n}{0.5^n + y^n} \right)\left((1-I) + I\frac{0.5^n}{0.5^n + x^n} \right) - y, \end{aligned} \tag{0.2}$$

where

$$k = \frac{a}{2\theta\delta}, \qquad b = \frac{b_y}{a}, \qquad S = \frac{b_x - b_y}{a}.$$

This reduces the system to the four-parameter model (n is fixed) utilized in the main text. In these terms k is a non-specific transcriptional control parameter, b is a basal rate of transcription, S measures the positional dependent asymmetry in the network (which is derived from cell contact for this model), and I is the strength of antagonism.

2.2 - Transcriptional Model Parameterization

Since this model has a relative complex parameter space, we first constrain it to a reasonable region. The strength of inhibition (*I*) can be constrained first by noting the size of the bistable region that supports distinct TE / ICM fates increases with strength (Figure Aa in S1 Text). A significant amount of antagonism ($I > 0.5$) is thus necessary. The strength of basal transcription (*b*) and the size of the contact mediated bias (*S*) are further constrained by expression data. We use four pieces of relative expression information from (Dietrich and Hiiragi, 2007) (at 55 hours post compaction, Fig. 4b): 1) Cdx2 is expressed at roughly 5-10 higher levels in TE cells than ICM cells, 2) Oct4 is expressed at 5-10 times higher levels in the ICM than TE, 3) the ratio of Oct4 / Cdx2 in ICM cells is ~5-10, and 4) the ratio of Cdx2 / Oct4 in TE cells is ~5-10. The former two provide relative inter-cellar expression information while the latter two provide relative intra-cellular expression information. Essentially, these are measures of how distinctive different cell types are.

We now seek regions of this four-dimensional parameter space that give rise to relative expression profiles in these ranges. Values are initially constrained to $I > 0.5$, $b<1$ (since above this value basal transcriptional processes outweigh auto-catalytic processes), and $b+S>0$ (to avoid negative transcription rates). The three-dimensional (b, S, I) parameter space is then sectioned in a grid, and for each parameter vector a bifurcation analysis of the system with respect to the remaining parameter *k* is performed (see Figure Ba for an example). For each parameter set $p=(b, S, I)$, we first distinguish between an interior ($S<0$) vs. exterior bias ($S>0$). For an interior (resp. exterior) parameter set *p*, the minimum and maximum values $k(p), K(p)$ that give rise to ICM (resp. TE) like fates is identified. See Figures C (a,b) where the blue and red dots respectively mark the bounds of the ICM / TE parameter regions.

In Figure Ab in S1 Text, the fraction of relevant *k* values that produce Oct4 / Cdx2 or Cdx2 / Oct4 expression ratios in the desired range for each parameter set *p* is indicated. For an interior set for example, we compute the quantity

$$f(p)=\frac{1}{K(p)-k(p)}\int_{k(p)}^{K(p)}\chi_{[5,10]}\left(\frac{O(k;p)}{C(k;p)}\right)dk,$$

where χ is a characteristic function, $O(k;p)$ is the Oct4 expression at that state, and $C(k;p)$ is the Cdx2 expression at that state. The colormap in Figure Ab in S1 Text indicates the value $f(p)$ for $I=0.6$; similar data for $I=0.7,0.8,0.9$ was produced but is not shown. This data constrains the parameter values that produce intra-cellular expression ratios in proper ranges.

We next considered inter-cellular expression relationships. Cdx2 and Oct4 expression respectively are compared pairwise between two model cells in ICM and TE states. Since the parameter ranges that produce ICM like states are more constrained than those that produce TE like states, for each pairing (I, b), the value of $S_{ICM}(I, b)<0$ that gives rise to the largest fraction of acceptable ICM states is chosen as a representative ICM parameter set. For each (I, b), we then search over all $S>0$ (which produce TE states). For each value of $S > 0$, 1000 values of *k* are drawn that produce both TE and ICM states (for $S > 0$ and S_{ICM} respectively). A thousand random pairs of ICM and TE states are then drawn pairwise and compared. The ratios

$$r_O = \frac{O(k_i, I, b, S_{ICM})}{O(k_j, I, b, S)}, \qquad r_C = \frac{C(k_j, I, b, S)}{C(k_i, I, b, S_{ICM})}$$

for each of the randomized 1000 parameter sets are computed, and the fraction of those tested parameter sets where both ratios are in the observed range are recorded in Figure Ac in S1 Text (for $I=0.6$).

From this data we draw the following conclusions. 1) An intermediate level of basal transcription (b) is required. Increasing (resp. decreasing) basal rates lowers (resp. raises) relative expression ratios. 2) As the transcriptional bias (S) becomes more extreme, expression ratios become larger, as expected. 3) As inhibition becomes stronger (increased I), expression ratios become more extreme. We chose an indicative parameter set based on these results that produce the observed expression ratios: $I = 0.6$, $k = 0.7$, $b = 0.7$, $S_{ICM} = -0.2$, $S_{TE} = 0.6$, which are indicated by white stars on Figures A (b,c) in S1 Text. These parameters are used to produce Figures 2-4, 6 in the main text. This is only a representative set used for in depth investigation and all results are shown hold for a substantial region of parameter space.

2.2.1 – Dependence of expression ratios on parameters.

Figures A (b,c) in S1 Text shows that as basal transcription rates decrease, the fraction of states that exhibit moderate expression ratios decreases. This results from an increase rather than a decrease in expression ratios. Essentially, as basal transcription rates decrease, the "off" value of expression (Oct4 expression in exterior cells for example) decreases to near 0 while the "on" value (Oct4 in interior cells for example) is relatively unaffected. This near exclusion of one factor leads to dramatically increased expression differences. As one would expect, increased strength of antagonism also increases expression ratios beyond the observed levels (results not shown), which again results primarily from exclusion of the "off" factor.

2.3 – Spatial Model Formulation

The multi-scale nature of this system lends itself to separate treatment of the intra-cellular gene regulatory network dynamics and biophysical spatial dynamics. We treat the former as a non-spatial stochastic system and endow each cell with a copy of a stochastic differential equation (SDE) representation accounting for the time evolution of transcription factor quantities. We use the relatively new sub cellular element method (SSEM) to model the spatial dynamics of the developing embryo. Using this, the following system dynamics are incorporated: 1) inter-cellular forces arising from contact and cell-cell adhesion interactions, 2) embryonic cavity formation (which occurs after compaction), 3) confinement of cells by the zona pellicuda, and 4) cell divisions. We provide a general overview of the sub-cellular element method and then separately describe the 2D disc and 3D spherical models. We note that the SSEM is not a "model" per se, but rather a modeling methodology much like agent based modeling or cellular potts modeling (CPM). Through previous investigation (Gord et al., 2014; Newman, 2005, 2008; Newman and Grima, 2004), this methodology has been shown to be particularly adept at describing multicellular systems where the physical shape, volume, and deformations are important and with proper assumptions can even re-capitulate rheological properties of cells (Sandersius and Newman, 2008). Given the small cell numbers and the potential for cell-cell interactions to be important in the developmental

process, we use this framework to describe the motions and interactions between cells. Gene regulation and fate specification are then modules that are layered on top of this physical model. We note that in the 3D (resp. 2D) model, cell fate determination is assumed to occur at the 16 (resp. 8) cell stage. Thus, the gene expression model discussed above only becomes active in simulations once sufficient numbers of cells are present. Prior to that, only the physical model is simulated.

2.4 - Sub-cellular element overview

The subcellular element method describes each individual cell as a discrete collection of subcellular "elements". Cell movements are driven by biomechanical force interactions including intracellular forces among elements of the same cell, intercellular forces between elements of different cells, and external interactions between the zona pellicuda, embryonic cavity, and cells. In this formulation, the ith cell is described by a collection of elements, which we index by $\{\alpha_i\}$. The position ($Y_{\alpha i}$) of these elements, whose dynamics are completely described by the sum total of their interactions with all other elements of the system, evolve according to a large Langevin system

$$\frac{dY_{\alpha_i}}{dt} = -\nabla_{\alpha_i} \sum_{\alpha_i \neq \beta_i} V_{intra}\left(\left|Y_{\alpha_i} - Y_{\beta_i}\right|\right) - \nabla_{\alpha_i} \sum_{i \neq j} \sum_{\beta_j} V_{inter}\left(\left|Y_{\alpha_i} - Y_{\beta_j}\right|\right) + F_{external}(Y_{\alpha_i}),$$

where V_{intra} is a pairwise potential energy between elements α_i and β_i of the same cell i, V_{inter} is a pairwise energy between elements α_i of cell i and β_j of cell j, and $F_{external}$ is any external force. Note this is an energetic formulation of this model, hence the negative gradient applied to the first two terms. Table B provides a list of all parameters used for this simulation.

2.5 - 2D disc model

For the 2D spatial model, each cell is represented by 40 elements. For each cell, these elements are grouped into two categories: 24 inner nodes and 16 external membrane nodes. All 40 nodes within a cell interact via an intra-cellular Morse potential

$$V(r) = U\exp\left(-\frac{r}{\xi}\right) - V\exp\left(-\frac{r}{\zeta}\right).$$

where $r = |Y_{\alpha_i} - Y_{\beta_i}|$. The combination of long range attraction and short range repulsion of this potential keeps the elements of each cell contained in a coherent structure and endows it with a preferred area. In addition to this Morse potential, each membrane node is attached via a spring force to its two adjacent neighbors, given by the spring potential

$$V = \mu\frac{(r - r_0)^2}{2}\hat{r},$$

where $\hat{r}$ is a unit vector in the direction of the connection between two elements and r_0 is a rest length. The Morse potential is also used to describe the force interactions between elements within different cells, but with different parameters.

2.5.1 - Cell Division

Each newly born cell is assigned a normally distributed cell cycle length. When this time expires, the mother cell is divided into two daughter cells by choosing a division plane (either randomly or in some informed way) and the cell is cut into two halves. A key

difference between this and the 3D model is that each cell in this model always maintains 40 elements. To accommodate this, upon division, each cell must be reinitialized with the proper number of elements. To do so, a contour is constructed from the remaining boundary elements of each daughter cell and these elements are subsequently replaced by 16 new boundary elements along that contour and the internal elements are replaced by 24 new elements, see Figure B in S1 Text.

Through this re-initialization process, the area of the embryo must remain fixed. So after division, the two daughter cells should be smaller than their mother. To accommodate the increased number of elements after division, we update the spatial scale of the system at discrete time points (Table A) to maintain a nearly constant embryonic volume.

2.5.2 – Embryonic Cavity Formation

Soon after compaction, a fluid filled embryonic cavity is known to form. Rather than account for the complexities of salt and osmotic transport, at the early 16 cell stage, a fixed phantom node is placed in the embryo (outside of all cells) that repels all other cells according to the Morse potential:

$$F_{external}(r) = \frac{U_{cavity}}{\xi} \exp\left(-\frac{r}{\xi}\right),$$

where r is again the distance between a node and the phantom node.

2.5.3 - Cell Contact and Signaling

Each cell is assigned a copy of the gene regulatory Equations 1.1 (main text) where cell contact influences dynamics by determining the value of S_i for each cell i. Rather than compute the fraction of external nodes that are currently bound to another cell, we instead compute the fraction of nodes that are in contact with the zona pellicuda. A critical assumption is made in this simplification that an interior cells interaction with the cavity is similar to cell – cell contact. We have found this necessary, since once the cavity forms, interior and exterior cells bordering the cavity are nearly indistinguishable from the standpoint of the fraction of cell – cell contact. Thus the contact based mechanism produces TE cells all around the periphery of the cavity. Using this method, we indirectly determine the fraction of boundary elements of a cell that are in contact with another cell and assign $S_i = 1.3 - 0.8 \cdot \mathit{ContactDensity}$. These values are chosen so that S_i ranges between the minimum and maximum values for interior / exterior determined in the parameterization section.

2.6 - 3D spherical model

In this model, in contrast to the 2D disc model, each cell is comprised of only one element type and all inter and intra cellular force interactions are described by Lennard–Jones type potentials

$$V = \varepsilon_{ij}\left(\left(\frac{\sigma_{ij}}{|r_{ij}|}\right)^{12} - \left(\frac{\sigma_{ij}}{|r_{ij}|}\right)^{6}\right),$$

where r_{ij} is the distance between element i and element j, the parameter ε_{ij} determines the strength of interaction, and σ_{ij} is the equilibrium separation where the inter-element potential is a minimum and no force is applied. In the absence of external forces, the

intra-cellular forces will scatter the inner elements to their rest lengths and the cell will always have a roughly spherical shape of preferred size. The full list of the parameters governing the inter and intra interactions between elements is provided in Table C.

2.6.1 – Cavity formation

We account for the formation of the embryonic cavity in much the same way as in the 2D model. Here, a single phantom element that repels other elements is placed at a fixed location within the embryo, with the repulsive force governed by

$$F_{external}(Y_{\alpha_i}) = -\nabla_{\alpha_i} \frac{\varepsilon_{external}}{|r_{\alpha_i}|},$$

where $r_{\alpha i}$ is again the separation between the phantom element and element α_{I} and the derivative is with respect to the position of element α_{I}. In this case however, we account more accurately for the nature of cavity formation in this system. Since the early embryo at this stage is a closed system, the formation of the cavity comes at the expense of cells losing volume. To account for this, the separation distance between elements is gradually decreased with time to mimic the shrinking of cells with time. The preferred element separation distance decreases directly as a function of cell numbers with the precise dependence shown in Table C.

2.6.2 - Cell division

A primary difference between this and the 2D disc model is that here, the total number of elements in the system is fixed for the entirety of the simulation and cell divisions simply subdivide them into daughter cells. Each cell is again assigned a normally distributed division time and when that time is reached, a plane in space is chosen to split the mother cell into two parts so that the resulting two daughter cells will have the same number of elements. So rather than maintaining a fixed number of elements per cell (as was the case in the 2D model), each division reduces the number of elements per cell by a factor of 0.5 (plus or minus one element).

3 – Model extensions and variants

3.1 – Cell division control

Given the hypothesized role of cell division control in organization (Gardner, 2001; Jedrusik et al., 2008; Johnson and Ziomek, 1981; Piotrowska-Nitsche and Zernicka-Goetz, 2005) and previously observed patterns of cell division in the developing embryo (Bischoff et al., 2008), we also investigated to what extent control over the orientation and type of cell division could direct accurate organization. To do so, we built on the base model (which we'll call Model 0 here) to incorporate different observations. First, in "Model 1", we incorporated the observed variability in cell cycle time by setting the standard deviation of division time to 15% (Bischoff et al., 2008).

In "Model 2" we incorporated symmetric versus asymmetric divisions into the model. Cells located in the interior of the embryo were forced to divide symmetrically (both daughter cells inherit the same fate as the parent) with a random division plane. Outer cells were allowed to divide either symmetrically or asymmetrically. For symmetric divisions, both daughters inherit the same fate as the parent and the division plane is chosen so that both daughter cells remain on the outside. For asymmetric

divisions, the division plane was chosen so that one cell remained on the outside and one on the inside, with the outside cell inheriting the TE (high Cdx2) and the inner cell automatically being assigned an ICM fate (high Oct4). The rationale for this treatment of asymmetric division is that asymmetric cell divisions are associated with polarity and it has been observed that prior to such a division, Cdx2 mRNA preferentially localizes to the apical side of the cell. Since an overabundance of asymmetric divisions of outside cells would lead to improper allocation between the TE and ICM lineages, we set the probability of an outside cell dividing asymmetrically to be 0.3.

One potential issue with Model 2 is that a fixed probability of different types of cell division could lead to improper allocation of cells to different lineages. Bischoff et al. (Bischoff et al., 2008) tracked the orientation of cell divisions over time and observed that the mode of division of a mother cell and its daughter cells were correlated. This suggests a possible compensatory mechanism for determining cell fates and allocation to different lineages. There they quantified the relative frequency of different types of cell divisions not just as a fixed quantity but as a function of time and ancestry. In "Model 3", we incorporated these observations. For the 1st cleavage, the division plane is chosen to be along the animal-vegetal axis. For 2nd cleavage, the probability of meridionally (M) and equatorially (E) oriented division planes for the two sister cells are ME (36%), EM (33%), MM (20%) and EE (11%). The division type for each sister cell is pre-assigned based on probability and recorded for further analysis. For the 3rd cleavage, there is no data so we assigned a random division orientation. For the 4th cleavage, the symmetric/asymmetric division ratio is 45/55. The mode of 5th cleavage is based on the division type of 4th cleavage generating each individual cell. We have no data for the 6th cleavage, so we imposed asymmetric division (division plane parallel to the embryo outer surface) for outer cells and symmetric division (division plane perpendicular to the embryo outer surface) for inner cells.

Following Figure 2b, we quantified the fraction of correct cells in the interior of the embryo as a function of gene expression noise size for Models 1-3 (Figure C in S1 Text). Results show that in all cases, noise is still required to achieve accurate organization. These results indicate that 1) while prepatterning and control over the mode of cell division have a possible role in organizing the embryo, something more is needed to ensure proper organization. And 2) noise at proper levels can ensure proper organization in each case.

3.2 – Location specific interactions of Cdx2 with DNA

Recent evidence (White et al., 2016) has suggested that interactions of transcription factors with DNA in cells of the developing blastocyst are different between inner and outer cells. In particular, it was shown that Cdx2 has longer residence times on DNA in outer cells and shorter residence times in inner cells. While we do not model the fine-grained details of protein / DNA interactions here, we can consider what the potential influence of this may be. On the one hand, this change in residence between inner and outer cells could differentially influence transcription of Cdx2 due to Cdx2 auto-regulation, which is already accounted for in the model (outer cells already exhibit higher Cdx2 transcription as a result of contact biases). Changes in residence time could alternatively influence the rate at which the protein is degraded. This could result if binding prevents post-translational modification of the targeted protein that leads to

degradation. To investigate this possibility, we consider an extension of this system accounting for potential differences in degradation ($\delta_x \neq \delta_y$).

$$x' = k\left(b + S + \frac{x^n}{0.5^n + x^n}\right)\left((1-I) + I\frac{0.5^n}{0.5^n + y^n}\right) - \delta_x x + \sigma_x x\sqrt{dw},$$
$$y' = k\left(b + \frac{y^n}{0.5^n + y^n}\right)\left((1-I) + I\frac{0.5^n}{0.5^n + x^n}\right) - \delta_y y + \sigma_y y\sqrt{dw}, \quad (0.3)$$

Based on (White et al., 2016), we assume that δ_x is higher (as a result of being unbound for longer times) in inner cells than outer cells. For simplicity, we fix *S=0* so that the only difference between inner and outer cells is the value of the Cdx2 degradation rate (δ_x).

Following the analysis in Figures 2(c,d), we assess how effective this mechanism would be at correcting mis-expressed cells that transit from an outer (Cdx2 positive) position to an inner position. To mimic this, we initialize 100 independent *in silico* cells in an "outer" cell state with (δ_x=0.5, the specific value of this initialization does not influence results provided it is <1). Simulations are run for a period of time to ensure each cell settles into a Cdx2+ steady state. Subsequently, the degradation rate is changed to a new value ($\delta_{x,inside}$) and simulations were run for an additional period of time under different noise conditions and the fraction of cells that correct their fate was assessed (Figure D in S1 Text).

Results show that without noise present, a sufficiently high level of Cdx2 degradation will lead to cell fate correction. This is to be expected since high degradation levels force Cdx2 to a low state, releasing inhibition of Oct4. When noise is included, the range of degradation levels that give rise to correction is increased. To compare with results of Figure 2f, we next assessed which noise source is responsible for cell fate corrections (where noise levels are necessary, see Figure Da in S1 Text for results). The dashed curves indicate scenarios where $\sigma_y = 0.3$, $\sigma_x = 0$ (mimicking noise in Oct4 only, gray dashed curve in Figure D in S1 Text) and $\sigma_x = 0.3, \sigma_y = 0$ (mimicking noise in Cdx2 only, black dashed curve). These results indicate that stochasticity in Cdx2 is more effective at driving cell fate corrections than stochasticity in Oct4.

3.2.1 – Spatial model with position dependent Cdx2 degradation.

We next augmented the 3D spatial model to include this alternative position sensing mechanism. To assess the capacity of this mechanism to direct organization, we first removed the position sensing mechanism discussed previously by setting *S=0* for all cells in the embryo at all time. This removes all contact biasing of cell fates. As with previous simulations, a single *in silico* cell goes through four divisions to give rise to a 16-cell embryo prior to turning on the gene expression model. Once reaching the 16-cell stage, the gene expression dynamics are initiated. In this model however, Cdx2 degradation rate is tied to cells position with $\delta_x = 0.5$ for outside cells (reflecting slower degradation) and $\delta_x = 1.2$ for inside cells (reflecting faster degradation). We again quantified (Figure Db in S1 Text) the accuracy of organization for different levels of stochasticity and found that, for this set of parameters, the embryo organizes accurately for sufficiently large inner cell degradation rates. Yet larger degradation rates yield accurate organization even without stochasticity.

We next incorporated both the original contact sensing (via the parameter *S*) mechanism along with this binding affinity based positional mechanism (via the parameter δ_x) into a single model (Figure Dc in S1 Text). Results show that the combined model yields accurate organization at the 64 cell stage and thus the two mechanism do not interfere with each other. Further comparison of Figures D (b,c) in S1 Text indicates that the combination of the two does lead to improved organizational accuracy (when compared to either individually) at the 32 cell stage. Thus each of these mechanisms has the potential to ensure robust and accurate organization of the embryo and in combination, they provide redundancies to further ensure organization proceeds correctly.

3.3 – Temporally dependent inhibition of Cdx2 by Oct4

While evidence indicates that Oct4 suppresses Cdx2 in ES cells (Niwa et al., 2005) and in mature blastocysts, evidence is less clear in the early blastocyst where Oct4 mutant embryos do not exhibit defects in Cdx2 localization (Ralston et al., 2010). Thus it appears that inhibition of Cdx2 by Oct4 may be temporally dependent, increasing over time. To assess the effect of this, we considered another variation of the underlying gene regulatory system where inhibition is not symmetric

$$x' = k\left(b + S + \frac{x^n}{0.5^n + x^n}\right)\left((1 - I_x) + I_x\frac{0.5^n}{0.5^n + y^n}\right) - \delta x + \sigma_x x\sqrt{dw},$$
$$y' = k\left(b + \frac{y^n}{0.5^n + y^n}\right)\left((1 - I_y) + I_y\frac{0.5^n}{0.5^n + x^n}\right) - \delta y + \sigma_y y\sqrt{dw}. \qquad (0.4)$$

Inhibition of Oct4 by Cdx2 (I_y) is held constant over time while inhibition of Cdx2 is gradually increased over time beginning at $I_x = 0$ at the 16 cell stage and linearly increasing to $I_x = 0.6$ at the 64 cell stage. Figure D in S1 Text shows the accuracy of organization as a function of time and noise strength. As with Figure 2b, results indicate that noise substantially improves the accuracy of organization by promoting cell plasticity.

3.4 – Cell fate biasing and the pre-patterning hypothesis

To this point, we have primarily investigated the efficacy of regulative hypotheses in organizing the embryo. There is evidence however to suggest that cell fates at later stages are biased by molecular heterogeneities between cells as early as the 2 or 4 cell stage (Gardner, 2001, 2005; Goolam et al., 2016; Piotrowska-Nitsche and Zernicka-Goetz, 2005; Tabansky et al., 2013). In particular, it was recently shown that at the 2 cell stage, Sox21 expression levels bias cell fates with a cells lower in Sox21 contributing slightly more cells to the TE than cells with higher Sox21 (Goolam et al., 2016). This is often referred to as the “pre-patterning” hypothesis.

We incorporated observations from (Goolam et al., 2016) to test to what extent such a molecular roadmap can direct organization in the absence of any positional based feedback regulation. To do so, we removed position based gene regulation from the 3D model and instead incorporated a pre-patterning mechanism (as discussed below) to determine cell fates.

For this model, we begin with a single cell that divides once to produce a 2 cell embryo. At this stage, we randomly assign one cell as high Sox21 and the other as low Sox21. This is not a modeled quantity but rather an assigned one that is inherited by all the daughter cells of each of these two blastomeres. From here, each cell undergoes two more divisions to produce an 8 cell embryo. At this point, TE and ICM fates have not been assigned since, in the embryo, clear fate determination appears to happen after compaction. At this stage, 4 cells carry the high / low Sox21 designation respectively. To proceed through subsequent cell division cycles and assign cell fates, we incorporate quantitative observations of cell division type and cell numbers from (Goolam et al., 2016).

There, the authors injected cells at the 2 cell stage with either a control siRNA or a Sox21 siRNA and tracked the progeny of those cells. They found that those injected with the control siRNA produced on average, 10.9 TE cells and 5.1 ICM cells, undergoing on average 4.4 asymmetric divisions in the process. Cells injected with Sox21 siRNA on the other hand produced ~13.7 TE cells and 2.1 ICM cells with an average of 2.1 asymmetric divisions. We associate the control data set with high Sox21 and the Sox21 siRNA data set with low Sox21 cells in our simulations and use this data to set the relative preference of each cell in the embryo to undergo (a)symmetric divisions as a function of the inherited Sox21 level.

To proceed from the 8 to the 16 cell stage, each cell probabilistically undergoes either a symmetric or asymmetric division. The probabilities for high and low Sox21 cells undergoing different divisions types are determined by the data above. In determining these probabilities, we account for the fact that inner cells are apolar and only outer cells divide asymmetrically. We have used a Monte Carlo procedure to determine the division type probabilities for each cell type that give rise to the appropriate number of asymmetric divisions. In the case of asymmetric divisions, the division plane is parallel to the embryo's surface, with the outer cell taking on a TE fate and inner cell an ICM fate. When outer cells divide symmetrically, the division plane is assumed to be perpendicular to the embryo surface, with both cells retaining their parent's fate. For inner cells, all divisions are symmetric with a randomly oriented division plane. By the 16 cell stage, each cell as either a TE or ICM fate and retains the high / low Sox21 designation of the parent cell. 16→32 and 32→ 64 cell divisions follow the same procedure.

Results (Figure Ea in S1 Text) indicate that organization is poor in this case. The central issue is that while this pre-patterning mechanism provides a correlation between Sox21 state at the 2 cell stage and cell fate at later stages, this and other pre-patterning hypotheses do not, to our knowledge, provide a mechanistic link between the early molecular heterogeneities and robust positioning of cells at later stages, which would be required to ensure proper organizing. We next built upon this model to again include the cell contact based position sensing mechanism discussed in the maintext. We find that the combined model does lead to proper organization (Figure Eb in S1 Text). These results suggest a likely scenario where early molecular heterogeneities bias cells to different fates, but an alternative mechanism ensures that cells have the ability to continually read their environment and change their fate accordingly to ensure proper organization.

4 – Sensitivity of model to parameters

Here we discuss the dependence of model results on parameters. This is a complex, multi-level model accounting for both physical and gene regulatory processes of the developing embryo. Given this complexity and the time required to simulate this model, an exhaustive analysis of the sensitivity of model results to parameters is not possible. We do however discuss here provide a partial analysis of the influence of each parameter (*b, I, k, δ)* on model results.

The question we primarily investigate in this paper is how cell-cell communication combined with stochastic effects promote organization of the embryo. We frame this analysis within these goals and determine how variations in different model properties influence the ability of cells to correct mis-specification errors and promote organization of the embryo.

We first note that in Figures 2(c,d) we investigated the influence of parameters representing the strength of basal transcription (b) and inhibition (I) on the capacity of cells to correct misspecification errors with and without noise. Results show that the region of parameter space where cells can correct errors expands with the presence of noise. This analysis does however assume that these parameters are the same for all cells in the embryo. We thus next ask how allowing differences in these parameters between cells influences in the embryo influences results. In the base model, the "effective" basal activation rate of Cdx2 is actually $b+S_i$. Thus complete symmetry of the basal activation rates is not required for noise to improve organization. Analysis of a model variant where inhibition of Cdx2 by Oct4 varies with time (but inhibition of Oct4 by Cdx2 is fixed with time, Figure E in S1 Text) shows the same results as the basic model, that noise improves organization. Thus, our central results hold when inhibition is asymmetric and are not highly sensitive to *I*.

In Figure D in S1 Text the influence of positionally dependent Cdx2 degradation rates (δ) was analyzed and similar results were found, stochasticity improves organization. Thus this results is not highly sensitive to the symmetry or precise values of degradation rates. Finally, we analyzed the influence of the parameter *k*, which is a scaling parameter for the total activation rate for Cdx2 and Oct4 by breaking the symmetry assumed in the original model and allowing Cdx2 and Oct4 to have different values of this parameter (denoted k_{Cdx2} and k_{Oct4}, Figure G in S1 Text). In this case we do find some sensitivity. If k_{Cdx2} is higher and k_{Oct4} is lower, stochastic effects cannot rescue organization. This is likely due to the fact that this combination increases the relative stability of the Cdx2 high state (relative to the base parameter set) while reducing the relative stability of the Oct4 high state. When k_{Cdx2} is lower and k_{Oct4} is higher, the system can organize accurately independent of the presence of noise. This is likely due to the lower relative stability of the Cdx2 high state ensuring cells exit this fate after relocation.

While these results do not constitute a full sensitivity analysis, they do suggest there will inevitably be a dependence of results on parameters, the basic supposition that stochastic effects improve the accuracy of organization is not highly sensitive to model parameters.

5 – Analysis of single cell gene expression data

Given the observations that expression variability of Cdx2 is higher than that of Oct4 we additionally analyzed a previous, independently produced qPCR data set (Guo et al.,

2010) quantifying single cell expression of 48 genes in the developing mouse blastocyst. Here, expression was collected at seven different developmental stages, i.e., 1-call (fertilized egg), 2-cell, 4-cell, 8-cell, 16-cell, ~32-cell, and ~64-cell stages. We considered data at the ~32 cell stage (when fate decisions are being made) and used a clustering (Marco et al., 2014) to identify cell types (ICM and TE) from this expression data. Subsequently, we quantified the coefficient of variation (CV) of Cdx2 and Oct4 in both ICM and TE cell populations (Figure J in S1 Text). We found that in the TE cell population, variability of Cdx2 and Oct4 were indistinguishable. In ICM cells however, Cdx2 exhibited significantly higher levels of variability. This data is not directly comparable to our immunofluorescence data since it represents variation over a population of embryos rather than within a single embryo. It is nonetheless consistent with our observations.

Tables:

Table A: Length scale updates for the 2D disk model after each "generation".

Generation	1	2	3	4	5	6
Cell Number	1	2	4	8	16	32
Length Scale (s)	1	0.8500	0.6672	0.4671	0.3036	0.1973

Table B: 2D model parameters. The scaling parameter *s* is from Table A in S1 Text.

Intra-Cellular Morse Potential			
$U = 0.5S$	$\xi = 1.2$	$V = 0.35s$	$\zeta = 2$
Intra-Cellular Membrane Springs			
$\mu = 100$		$r_0 = 0.7023s$	
Inter-Cellular Morse Potential			
$U = 0.08$	$\xi = 0.06$	$V = 0.01$	$\zeta = 0.08$
Cell-Boundary Morse Potential			
$U = 0.7S$	$\xi = 0.2s$	$V = 0.55s$	$\zeta = 0.5s$
Cavity Morse Potential			
$U_{cavity} = 0.008$		$\xi = 1.5$	
Other Parameters			
Number of membrane nodes in each cell			16
Number of inner nodes in each cell			24
Total number of nodes in each cell			40
Time step to update mechanical ODE's			$dt_{\text{mech}} = 0.01$
Time step to update signaling ODE's			$dt_{\text{signal}} = 0.1$
Embryo boundary			$R = 3.15$

Table C: 3D spatial model simulation parameters.

**Parameters**	**Value**
Element number	640
Embryo radius	10
Division cycle	10000 time steps
Intra-cellular potential	ε_{ij} =1.5, σ_{ij} = 2/(1+0.1$\log_2 N$), no cut-off distance. (N is cell number)
Inter-cellular potential	ε_{ij} =0.05, σ_{ij} = 4/(1+0.1$\log_2 N$), cut-off distance is 10.0.
External force	$\varepsilon_{external}$ =0.001
Time step	dt = 0.1

Table D: Table of antibodies used for this study which includes, dilutions used, supplier, catalog #, and lot # information

**Antibody**	**Dilution used**	**Supplier**	**Catalog #**	**Lot #**
Rabbit anti-Oct4 (C-10)	1:250	Santa Cruz Biotechnology Inc.	sc-9081	G0607
Mouse Anti-Cdx2	1:200	BioGenex	MU392A-UC	MU392A0114X
Goat anti-rabbit (Alexa-555)	1:200	Life Technologies	A21428	1511349
Goat anti-mouse CFL 647	1:200	Santa Cruz Biotechnology Inc.	Sc-362287	A0912

Figures:

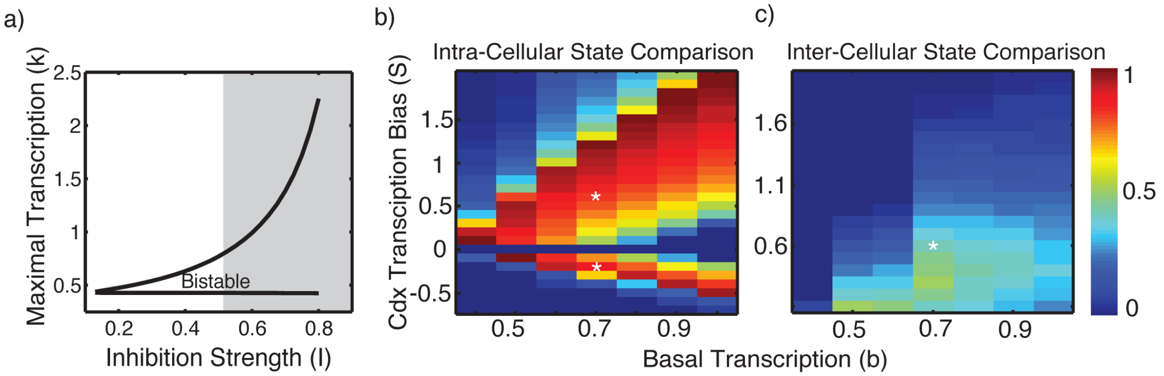

Figure A: ***Panel a)*** Size of the bistable parameter regime (with respect to the transcriptional parameter *k*) as a function of the strength of competitive inhibition *I*. Further parameter searches are restricted to the shaded region, which emits a substantial bistable parameter regime. *b=0.7, S=0, I=0.6, n=4*. ***Panel b)*** The fraction of transcriptional states (for *k*) that yield Oct4 – Cdx2 expression relationships in the proper ranges (e.g. 5-10) for the respective inner and outer cell fates. White stars represent parameter values used in future simulations. ***Panel c)*** Fraction of randomly chosen pairs of representative ICM and TE cell states that produce Cdx2 and Oct4 ratios in the observed ranges. See SM text for simulation methods and further information.

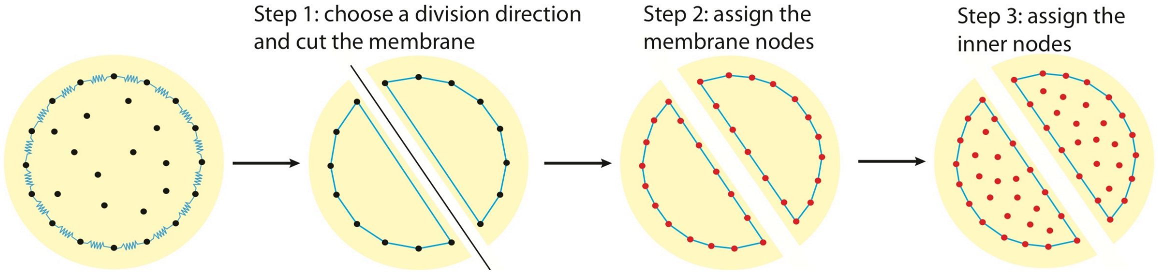

Figure B: Schematic of the cell division process in the 2D model. First, a division plane is chosen to separate the elements into equal subsets. Second, the original membrane nodes are replaced with 16 new membrane nodes. Finally, the interior of each new cell is populated with 24 interior nodes, resulting in two new cells.

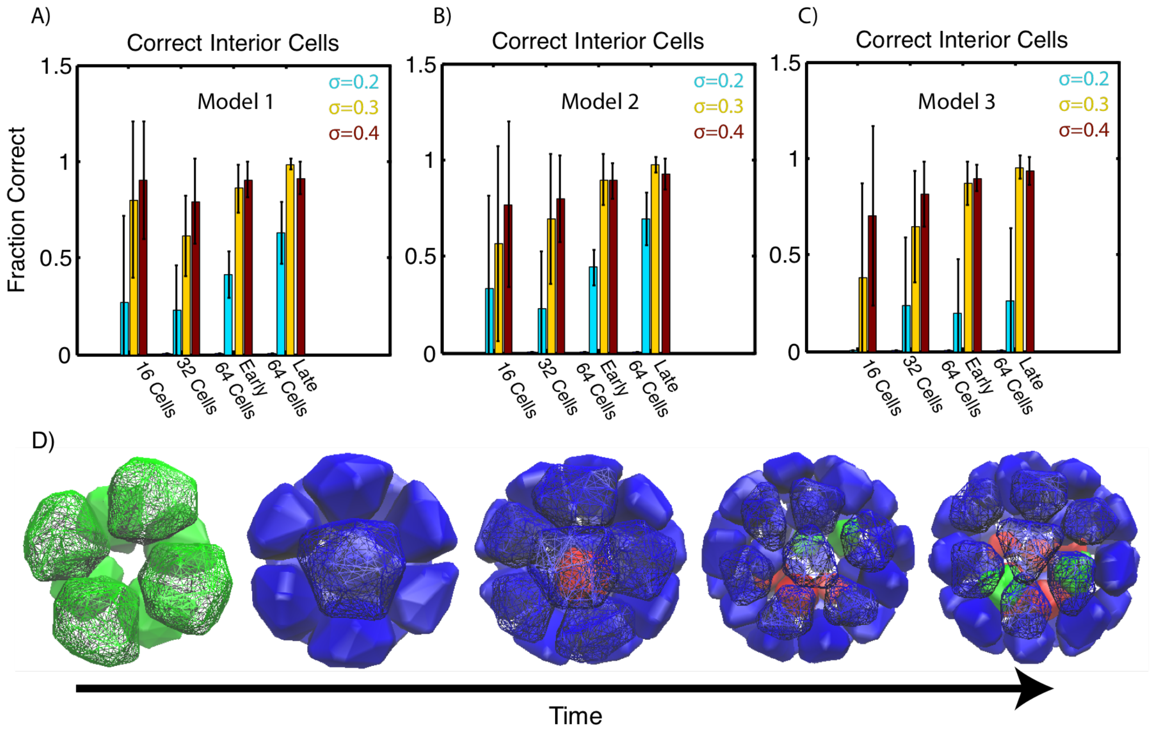

Figure C: Influence of polarity and cleavage / division control on organization. ***Panels A-C***) The effectiveness of organization for three model variants was quantified to assess the efficacy of different assumptions on the mode of cell division. Results indicate the fraction of correct interior cells, which is a surrogate measure for the accuracy of organization, as a function of noise amplitude. An ensemble of 20 simulations is again used to account for simulation randomness. The methods and plotting conventions are the same as those used to produce Figure 2b. The model used to generate Figure 2b could be thought of as Model 0 on top of which these adjustments to the type and orientation of cell division were made. As with Figure 2b, accuracy is poor when no stochasticity is present ($\sigma=0$). ***Panel D***) Snapshots showing the time evolution of Model 2 in the low noise regime ($\sigma=0.2$). Plotting conventions are the same as in Figure 5b: blue, red, and green represent Cdx2 dominant, Oct4 dominant, and mixed expressions, respectively. It is not visually evident due to the geometry, but a number of interior cells at the end are Cdx2 dominant.

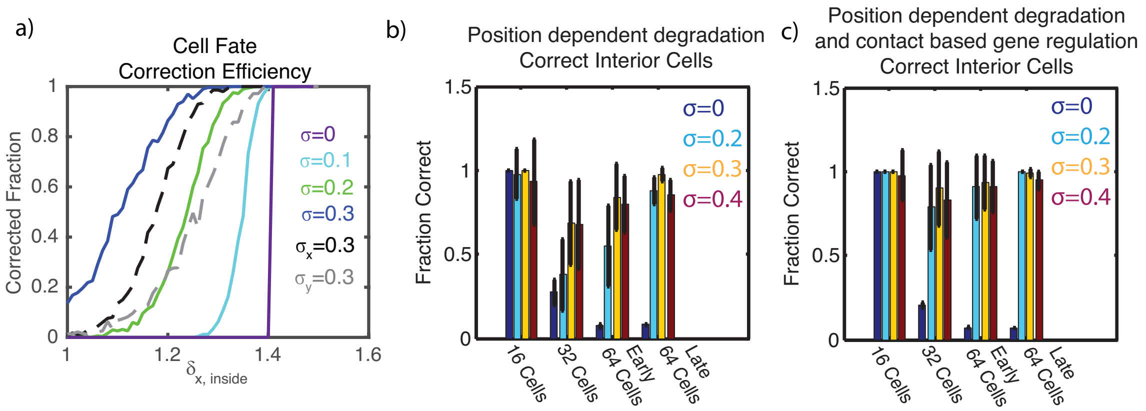

Figure D: Location specific interactions of Cdx2 with DNA. ***Panel a)*** Cell fate correction efficiency as a function of the change of degradation rate upon relocation from the outside to the inside of the embryo. Cells are initiated in a state with $\delta_x = 0.5$. Relocation is mimicked by adjusting this value to $\delta_x = \delta_{x,inside}$ and simulations are continued. Results depict the fraction of cells that switch from the Cdx2+ to the Oct4+ state. The purple, turquois, green, and blue curves respectively look at the system with isotropic noise levels. For the gray dashed curve $\sigma_x = 0$ and for the black dashed curve $\sigma_y = 0$. The bias parameter is fixed at *S=0* for the entirety of the simulation. All other parameters are as listed in SM section 2.2. ***Panel b)*** Simulation of the full spatial model with *S=0* (contact bias removed completely) and position dependent Cdx2 degradation rates. See Section 3.2.1 for model specifics. ***Panel c)*** Simulation of the full spatial model with both contact based Cdx2 transcription bias and position dependent Cdx2 degradation rates. In (b,c), plotting conventions are the same as described in Figure 2b of the main text.

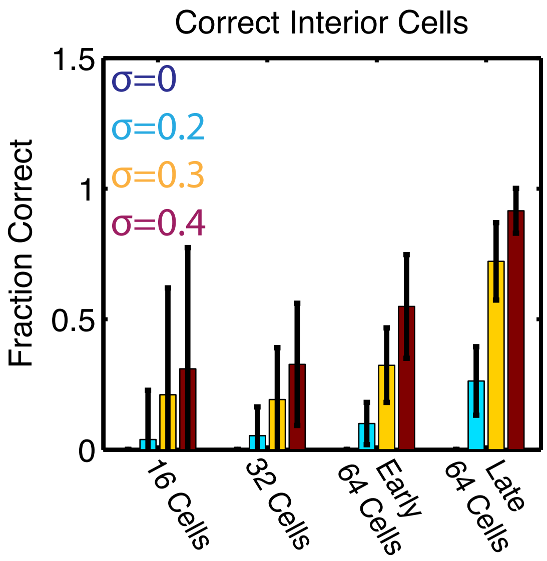

Figure E: Temporally dependent inhibition of Cdx2 by Oct4. Accuracy of organization as a function of noise strength and time (See Figure 2b for plotting conventions) for the model with gene regulation as in SM Eqs. 0.4. In this simulation, the value of I_x linearly increases from 0 to 0.6 between the 16 cell and 64 cell stages to mimic temporally increasing strength of inhibition. All other parameters and simulation specifics are identically as in Figure 2b.

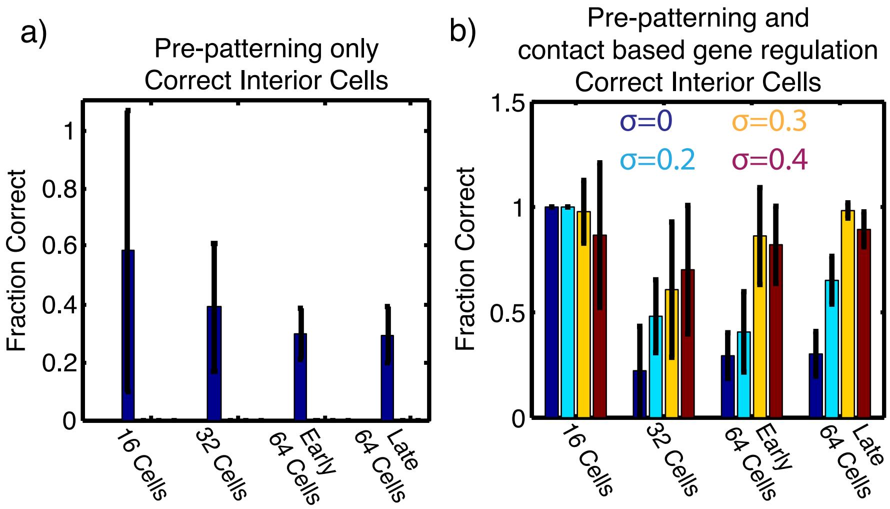

Figure F: Pre-patterning model. Accuracy of organization as a function of time and noise strength (in b). ***Panel a)*** The 3D spatial model is simulated with an imposed Sox21 prepattern at the 2 cell stage determining the frequency of subsequent cell division types. The Oct4 / Cdx2 gene regulation model is removed to determine the efficacy of the prepatterning hypothesis in isolation. For this reason, there are no noise levels to consider and only a single bar is presented at each time point. ***Panel b)*** The pre-patterning model, which biases cell fate and division type decisions, is combined with the contact based gene regulation model. See Figure 2b for plotting conventions.

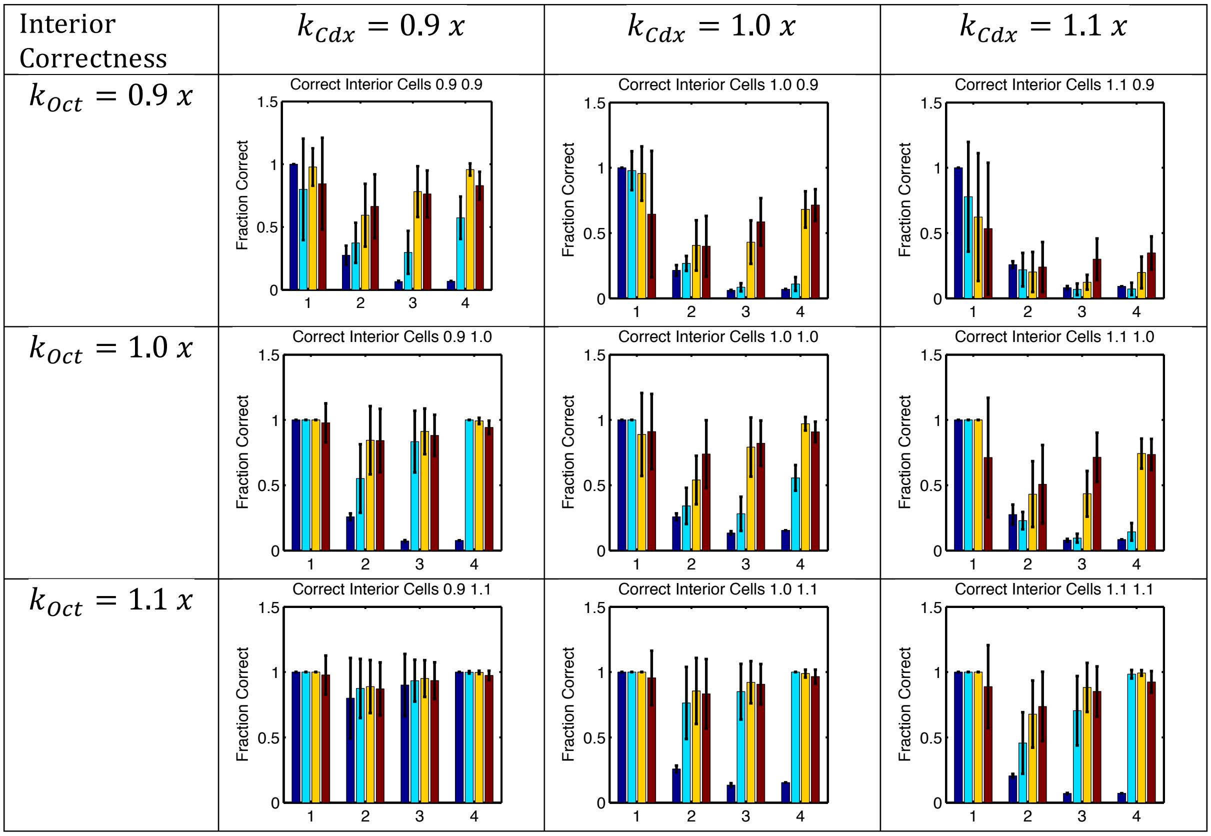

Figure G: Sensitivity to "k". In the base gene regulation model Eqs. (0.2), the value of the parameter "k" is taken to be the same for both Cdx2 (variable x) and Oct4 (variable y). Here we assess sensitivity of our results to this assumption. The base value of this parameter is $k=0.7$ (see SM Section 2.2). Here, we allow Oct4 and Cdx2 to have separate values of this parameter, denoted here as k_{Oct4} and k_{Cdx2}. Each tile in this table shows accuracy results (plotting conventions are the same as in Figure 2b) for different pairings of these values. The notation *0.9 x* or *1.1* x and so forth indicates a 0.9 or 1.1 fold change (e.g. 10% change up or down) from the base value for "k". Results indicate that when $k_{Oct4} < k_{Cdx2}$, accuracy is impaired.

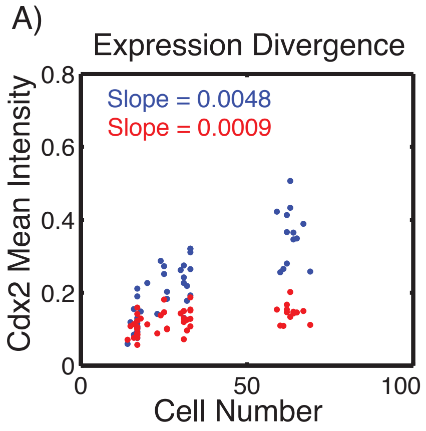

Figure H: Replication of results. Replication of Figure 5a. Cdx2 intensity versus ``embryo age'' as measured by cell number (50 Embryos, min = 16, max = 64, mean = 33 cells). Each data point represents the average normalized intensity of Cdx2 in *ICM* (red) or *TE* (blue) cells of a single, fixed embryo. Slopes of the best-fit regression line are provided with p=1e-13 (blue) and p=1e-4 (red), indicating both exhibit a significant positive correlation with cell number. This data comes from whole cell segmentation rather than nuclear segmentation. Raw data is normalized by DNA marker intensity, however, qualitative results are similar with either raw or cell volume normalizations.

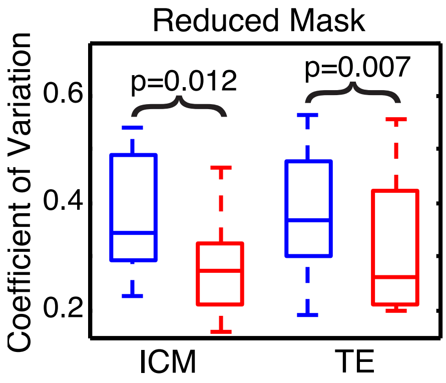

Figure I: Re-segmentation of data in Figure 3f. All 10 embryos measured in Figure 3f were resegmented using different active contour parameters that produce smaller masks (see SM text 1.1). All coloring and conventions are the same as in Figure 3f.

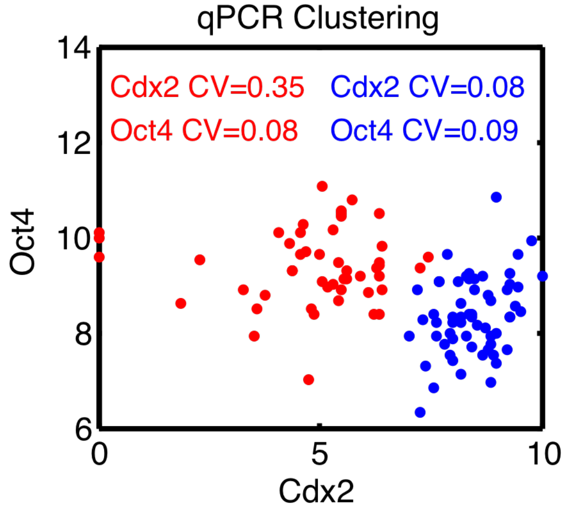

Figure J: Analysis of qPCR expression data: Cluster analysis was performed on single cell qPCR expression data of 109 cells isolated from a total of 8 embryos, at the ~32 cell stage (not all cells from each embryo were successfully harvested). Results show expression of Oct4 and Cdx2 in ICM (red) and TE (blue) cells. Coefficient of variation for each transcription factor in each population is provided. Note that coefficient of variation is significantly higher for Cdx2 than Oct4.

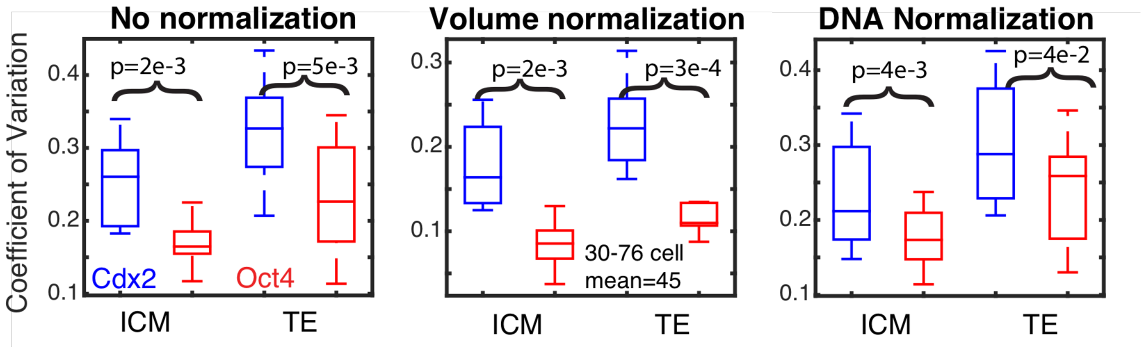

Figure K: Replication of noise quantification with swapped secondary antibodies: To check whether specific antibodies used in immunofluorescence are responsible for the asymmetry observed in Figure 3f, we replicated this experiment with swapped secondary antibodies. 9 embryos ranging from 30-76 cells in size were imaged and segmented. To ensure the method of fluorescence normalization does not impact the observation that Cdx2 variability is higher than Oct4 variability, this analysis was performed with 1) the raw data (no normalization), 2) data normalized by the quantified cell volume, and 3) data normalized by DNA content (Hoechst stain). Compare results to Figure 3f.

References:

Bischoff, M., Parfitt, D.E., and Zernicka-Goetz, M. (2008). Formation of the embryonic-abembryonic axis of the mouse blastocyst: relationships between orientation of early cleavage divisions and pattern of symmetric/asymmetric divisions. Development *135*, 953-962.

Christley, S., Lee, B., Dai, X., and Nie, Q. (2010). Integrative multicellular biological modeling: a case study of 3D epidermal development using GPU algorithms. BMC Systems Biology *4*.

De Solorzano, C.O., Malladi, R., Lelievre, S.A., and Lockett, S.J. (2001). Segmentation of nuclei and cells using membrane related protein markers. J Microsc-Oxford *201*, 404-415.

Dhooge, A., Govaerts, W., and Kuznetsov, Y.A. (2003). Matcont: A Matlab Package for Numerical Bifurcation Analysis of Odes. Acm T Math Software *29*, 141-164.

Dietrich, J.E., and Hiiragi, T. (2007). Stochastic patterning in the mouse pre-implantation embryo. Development *134*, 4219-4231.

Gardner, R.L. (2001). Specification of embryonic axes begins before cleavage in normal mouse development. Development *128*, 839-847.

Gardner, R.L. (2005). The case for prepatterning in the mouse. Birth defects research Part C, Embryo today : reviews *75*, 142-150.

Goolam, M., Scialdone, A., Graham, S.J., Macaulay, I.C., Jedrusik, A., Hupalowska, A., Voet, T., Marioni, J.C., and Zernicka-Goetz, M. (2016). Heterogeneity in Oct4 and Sox2 Targets Biases Cell Fate in 4-Cell Mouse Embryos. Cell *165*, 61-74.

Gord, A., Holmes, W.R., Dai, X., and Nie, Q. (2014). Computational modelling of epidermal stratification highlights the importance of asymmetric cell division for predictable and robust layer formation. J R Soc Interface *11*.

Guo, G., Huss, M., Tong, G.Q., Wang, C., Li Sun, L., Clarke, N.D., and Robson, P. (2010). Resolution of cell fate decisions revealed by single-cell gene expression analysis from zygote to blastocyst. Developmental cell *18*, 675-685.

Humphrey, W., Dalke, A., and Schulten, K. (1996). VMD: visual molecular dynamics. Journal of molecular graphics *14*, 33-38, 27-38.

Jedrusik, A., Parfitt, D.E., Guo, G., Skamagki, M., Grabarek, J.B., Johnson, M.H., Robson, P., and Zernicka-Goetz, M. (2008). Role of Cdx2 and cell polarity in cell allocation and specification of trophectoderm and inner cell mass in the mouse embryo. Genes & development *22*, 2692-2706.

Johnson, M.H., and Ziomek, C.A. (1981). The foundation of two distinct cell lineages within the mouse morula. Cell *24*, 71-80.

Marco, E., Karp, R.L., Guo, G., Robson, P., Hart, A.H., Trippa, L., and Yuan, G.C. (2014). Bifurcation analysis of single-cell gene expression data reveals epigenetic landscape. Proceedings of the National Academy of Sciences of the United States of America *111*, E5643-5650.

Newman, T.J. (2005). Modeling Multicellular Systems Using Subcellular Elements. Mathematical Biosciences and Engineering *2*, 611-622.

Newman, T.J. (2008). Grid-Free Models of Multicellular Systems, with an Application to Large-Scale Vortices Accompanying Primitive Streak Formation. Current Topics in Developmental Biology *81*, 157-182.

Newman, T.J., and Grima, R. (2004). Many-body theory of chemotactic cell-cell interactions. Physical Review E *70*.

Niwa, H., Toyooka, T., Shimosato, D., Strumpf, D., Takahashi, K., Yagi, R., and Rossant, J. (2005). Interaction between Oct3/4 and Cdx2 determines trophectoderm differentiation. Cell *123*, 917-929.

Piotrowska-Nitsche, K., and Zernicka-Goetz, M. (2005). Spatial arrangement of individual 4-cell stage blastomeres and the order in which they are generated correlate with blastocyst pattern in the mouse embryo. Mech Develop *122*, 487-500.

Ralston, A., Cox, B.J., Nishioka, N., Sasaki, H., Chea, E., Rugg-Gunn, P., Guo, G., Robson, P., Draper, J.S., and Rossant, J. (2010). Gata3 regulates trophoblast development downstream of Tead4 and in parallel to Cdx2. Development *137*, 395-403.

Sandersius, S.A., and Newman, T.J. (2008). Modeling cell rheology with the Subcellular Element Model. Physical biology *5*, 015002.

Tabansky, I., Lenarcic, A., Draft, R.W., Loulier, K., Keskin, D.B., Rosains, J., Rivera-Feliciano, J., Lichtman, J.W., Livet, J., Stern, J.N.*, et al.* (2013). Developmental bias in cleavage-stage mouse blastomeres. Current biology : CB *23*, 21-31.

White, M.D., Angiolini, J.F., Alvarez, Y.D., Kaur, G., Zhao, Z.W., Mocskos, E., Bruno, L., Bissiere, S., Levi, V., and Plachta, N. (2016). Long-Lived Binding of Sox2 to DNA Predicts Cell Fate in the Four-Cell Mouse Embryo. Cell *165*, 75-87.
